# Clinical Trajectories in Adolescents with and without a History of Non-Suicidal Self-Injury: The BRIDGES Longitudinal Study

**DOI:** 10.20900/jpbs.20230007

**Published:** 2023-07-01

**Authors:** Aparna U. Nair, Julia A. Brekke-Riedl, Michaelle E. DiMaggio-Potter, Katherine A. Carosella, Carolyn Lasch, Rylee Brower, Victoria Papke, Kristina Reigstad, Bonnie Klimes-Dougan, Kathryn R. Cullen

**Affiliations:** 1 Department of Psychiatry and Behavioral Sciences, University of Minnesota, Minneapolis, MN 55454, USA; 2 Medical Scientist Training Program, University of Minnesota, Minneapolis, MN 55455, USA; 3 Department of Psychology, University of Minnesota, Minneapolis, MN 55455, USA; 4 Institute of Child Development, University of Minnesota, Minneapolis, MN 55455, USA

**Keywords:** non-suicidal self-injury, adolescents, development, depression, suicide risk

## Abstract

**Background:**

Non-suicidal self-injury (NSSI) is a highly prevalent clinical concern in adolescents and is associated with impaired functioning and suicide risk. The BRIDGES (BRain Imaging Development of Girls’ Emotion and Self) study was designed to collect longitudinal clinical and neurobiological data to advance our understanding of NSSI in adolescents. The purpose of this paper is to describe the clinical data collected as part of this study, including psychiatric diagnoses, depression symptoms, episodes of non-suicidal self-injury, suicidal thoughts and behaviors, childhood trauma, and personality domains.

**Methods:**

The baseline sample included 164 adolescents aged 12–16 assigned female at birth (Mean age = 14.97, SD = 1.20) with NSSI histories ranging from none to severe. Participants and their parent/guardian were invited to provide data at three time points spaced approximately one year apart. Descriptive analyses were conducted to provide estimates of rates and trajectories of clinical data.

**Results:**

Of the 164 study participants, 75.61% and 57.93% completed the second and third time points, respectively. Visual inspection of the data suggests an overall trend of decreasing severity of psychopathology over time, and adolescents with a history of NSSI appeared to have higher rates of psychopathology than those without.

**Conclusions:**

This paper describes longitudinal clinical trajectories in adolescents with a range of NSSI histories and presents readers with an overview of the rich, publicly available dataset that we hope will inspire future research to advance the understanding of the neurodevelopmental trajectories associated with NSSI, depression, and suicide risk.

## INTRODUCTION

Non-suicidal self-injury (NSSI) is a problematic transdiagnostic behavior that is increasingly common in adolescents and is associated with an increased risk for future suicide attempts [1,2]. Prevalence rates in adolescents range from 7.5 to 46.5% [3]. While longitudinal research is still emerging, the extant evidence suggests that rates of NSSI increase during the adolescent period (typically in early to mid-adolescence) and then decrease in early adulthood [4]. There has been a call for more longitudinal research, especially to address gaps in knowledge about the neurobiological trajectories underlying the clinical course of NSSI in adolescents [5,6]. In addition, there is a need to characterize how clinical characteristics evolve across adolescence.

In adolescents, NSSI typically occurs in the context of emotional distress as a maladaptive strategy to regulate one’s negative affect [7]. This behavior commonly co-occurs with other clinical problems, such as depression, anxiety, and suicidal ideation, and, like many other clinical problems, is frequently preceded by a history of adverse experiences [8–10]. The shared and yet distinct nature of the precursors, correlates, and outcomes of overlapping problems like NSSI, depression, and suicidal ideation and behavior provides guiding clues for shared vulnerabilities and introduces a challenge for research [11]. Additionally, NSSI has been related to mania symptoms and diagnosis of bipolar disorder [12] and borderline personality disorder [13]. However, current knowledge is mostly based on cross-sectional studies, with scarce research on the longitudinal trajectory of these phenomena [4,8,10]. Overall, there is a need for longitudinal research to characterize the developmental trajectories of these co-occurring phenomena in order to better understand the complex course of NSSI in youth.

We recently conducted a longitudinal study funded by the National Institutes of Mental Health (NIMH), examining NSSI in adolescents with and without a history of NSSI. The BRIDGES (BRain Imaging Development of Girls’ Emotion and Self) study was designed to advance the NIMH Research Domain Criteria (RDoC) initiative, which aims to create a biologically-based framework for understanding psychiatric disorders [14]. To this end, we collected longitudinal, multilevel data within three domains that are relevant to NSSI: Sustained Threat [15], Cognitive Control [16], and Self Knowledge (Thai et al., in preparation). The primary goal of this study was to examine changes within these RDoC domains across adolescence and how aberrant patterns of development in these domains may map onto psychopathology and suicide risk. To ensure a range of NSSI severity, recruitment sought to represent four categories: No NSSI (+/− psychiatric diagnoses), Mild NSSI (<4 lifetime NSSI episodes), Moderate NSSI (5 or more episodes, frequency <1 per month) and Severe NSSI (frequency > 1 per month). Data are publicly available through the National Data Archive (NDA).

The goal of the current paper is to describe the longitudinal clinical data from the BRIDGES sample. In particular, we focus on mental health diagnoses, depression symptoms, NSSI thoughts and behaviors, suicidal thoughts and behaviors (STBs), childhood trauma, and personality domains. While adding to current knowledge about the clinical presentation of adolescents with NSSI and how NSSI and related concerns evolve over the adolescent period, we aim to raise awareness of the richness that this public dataset has to offer. We hope that these detailed results will spark ideas for researchers to formulate and pursue their own questions about neurobiological mechanisms and neurodevelopment with these data. We also discuss the challenges of retaining this high-risk population in research studies that require a high burden of assessments.

## MATERIALS AND METHODS

### Ethical Oversight

The study was approved and overseen by the University of Minnesota Institutional Review Board (IRB #1605M88102; approval date: 07th July 2016).

### Sample Definition, Recruitment, Screening, and Consent

Inclusion criteria for entering the study were: (1) age 12–16 years; (2) identified as female sex at birth; (3) already had first menses; (4) Magnetic Resonance Imaging (MRI) compatible; (5) no history of an intellectual disorder; (6) no current substance use disorder (other than nicotine use); (7) no history of a primary psychotic disorder; (8) no history of bipolar spectrum disorder; (9) no autism spectrum disorder; (10) no major medical illness; (11) no pregnancy; (12) willing to have deidentified data shared with RDoC database; (13) capacity to consent based on UCSB Brief Assessment of Capacity to Consent; (14) English speaker. We recruited these adolescents into one of four severity categories: No NSSI (no prior NSSI history), Mild NSSI (Fewer than 4 episodes of NSSI that involved tissue damage), Moderate NSSI (At least 4 episodes of NSSI that involved tissue damage, frequency < 1/month) and Severe NSSI (At least 4 episodes of NSSI that involved tissue damage, frequency ≥ 1/month). Only adolescents assigned female at birth were recruited due to the higher prevalence of NSSI in females [17]. Recruitment outreach included advertisements that were distributed through schools, clinics, and social media. When parents expressed interest in having their children participate in the study, they completed a telephone screening process.

**Figure 1.**
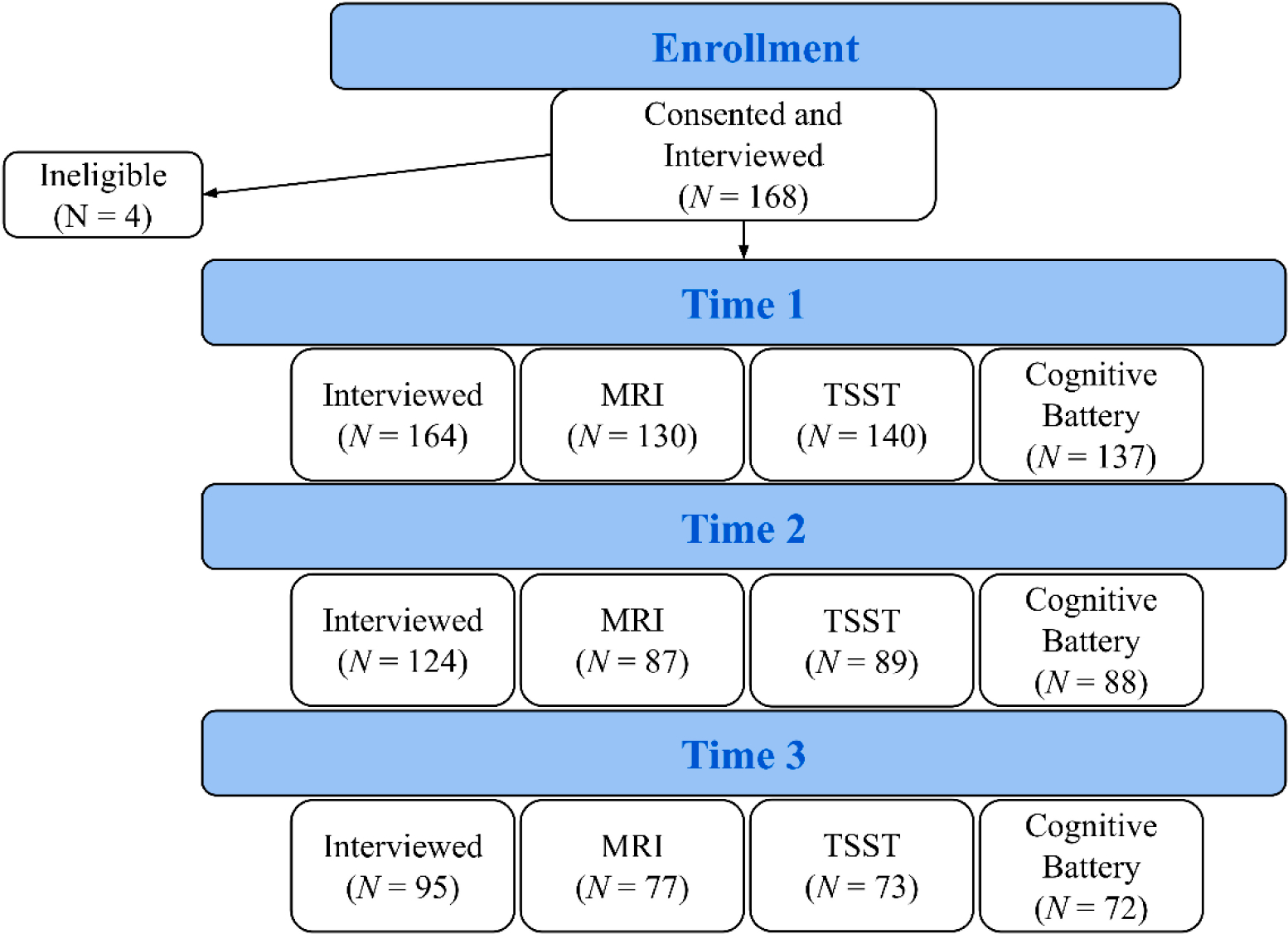
Consort Diagram and Timeline of Visits. *Note:* The number of participants who completed at least one type of visit at T1, T2, and T3 are 164, 124, and 95, respectively. Of the 164 participants who completed the T1 interview, 40 did not complete any T2 visits, and 69 did not complete any T3 visits. MRI = Magnetic Resonance Imaging, TSST = Trier Social Stress Test.

Participants who met initial eligibility were scheduled for an in-person consent visit (in which parents completed the signed consent and adolescents who were minors completed a signed assent), which was followed by a clinical assessment. Beginning in March 2020, the consent and clinical evaluation process shifted from in-person to Zoom, with consent forms signed electronically. Following their initial clinical assessment, participants were invited for two additional visits to collect cognitive/neurophysiological and neuroimaging data. Additionally, participants were invited to return one and two years after their initial visits (see Figure 1 for a timeline of visits). In some cases, the gap between visits was longer than expected due to numerous factors, including the COVID-19 pandemic. For this reason, we refer to the visits as Time 1, Time 2, and Time 3 (T1, T2, and T3). On average, there was a gap of 1.21 (SD = 0.26) years between clinical assessment visits at T1 and T2 and a gap of 1.21 (SD = 0.39) years between clinical assessment visits at T2 and T3. Several of the measures described below were assessed at more than one of these timepoints in order to examine changes in psychopathology over time. For these measures, participants were provided with the same questions at every timepoint. Generally, measures were based on past weeks, months, or years. Occasionally (e.g., CTQ), the measures assessed lifetime symptoms at baseline and once again assessed lifetime symptoms at T2 and T3. Data collection lasted approximately six years, from December 2016 to July 2022. Participants were paid for contributing their time to this study.

### Clinical Assessment

#### Safety

For all clinical assessments, using all of the information detailed below as a starting point, our protocol included a detailed procedure for identifying, assessing, and responding to suicide risk, including (in collaboration with participants and their parent/guardian) safety planning and making appropriate referrals. When participants indicated suicidal ideation or a significant increase in self-harm behaviors, the research team followed up with the participant to further assess risk. This was done by the research staff under the direct supervision of an on-call clinical investigator (KRC, BKD, and/or KR), or it was done directly by the clinician. The research team engaged in conversation with the participant to clarify the level of risk (i.e., additional details on recent thoughts and behaviors, level of current suicide intent, and details on suicide plans, if any). Parents/guardians were involved in the conversation if the team perceived significant suicidal ideation with intent and/or plan. The research team engaged the participant and parent/guardian in safety planning and provided referrals for mental health intervention when warranted. In our protocol, participants with severe self-harm that required stitches or hospitalization or who endorsed suicidal ideation with intent and did not agree to a safety plan would be referred to the Emergency Room. Fortunately, none of the participants in our study required this level of intervention.

#### Diagnosis

Diagnosis was determined using the Kiddie Schedule for Affective Disorders and Schizophrenia for School-Age Children-Present and Lifetime Version (K-SADS-PL) [18]. This is a well-validated interview, with excellent interrater reliability (93–100%) and good to excellent test-retest reliability (κ = 0.63–1.00). The first 39 cases at T1 completed the paper copy of the K-SADS, and the subsequent participants completed the online K-SADS. Diagnoses were based on consensus between parent and adolescent reports using the K-SADS algorithm as a guide. All diagnoses underwent a final review in supervision with the licensed supervisors (BKD, KR), resulting in occasionally altered diagnoses. Issues relevant to safety were addressed, and referrals were provided to families when needed.

#### Depression symptom severity

Depression symptom severity was assessed using the self-report Beck Depression Inventory-II (BDI-II) [19] and the clinician-administered Children’s Depression Rating Scale, Revised (CDRS-R; adolescent and parent) [20]. The BDI-II consists of 21 items with a high internal consistency (coefficient alpha = 0.91) and otherwise well-established validity and reliability in adolescents and clinical populations [21]. The CDRS-R interview consists of 17 questions, 3 of which are behavioral observations of the child and are omitted during the parent interview, and has good interrater reliability (*r* ≥ 0.81) [22]. In adolescent samples, it has been shown to have good internal consistency (Cronbach’s α ≥ 0.74) and to correlate with other measures of depression (e.g., K-SADS) [23].

#### Self-injurious thoughts and behaviors

Self-Injurious Thoughts and Behaviors were assessed using several measures. The Self-Injurious Thoughts and Behaviors Interview (SITBI) [24] was conducted with the adolescent. This interview captures the presence, frequency, severity, and characteristics of the reporter’s suicidal ideation, attempts, plans, gestures, and non-suicidal self-injury (although, due to its low reliability, we did not complete the gesture scale in this study). The SITBI has strong test-retest reliability, interrater reliability, and concurrent validity with other measures of NSSI and suicidality. Included in this dataset are the age of first, age of last, estimation of approximate number of NSSI thoughts and engagement in the past week, month, and year as well as the intensity of the NSSI thoughts. Additionally, participants were inquired about potential reasons and motivations for NSSI thoughts and engagements, which will be discussed in detail in this paper.

As noted previously, the SITBI was also used to categorize participants into one of four groups based on NSSI severity: No NSSI, Mild NSSI, Moderate NSSI, and Severe NSSI. Criteria for group assignment (described in Table 1) were intended to capture a range of severity of NSSI history in terms of both the number of episodes and the severity of injury. There is currently no widely accepted, standard approach to assessing NSSI severity. While acknowledging that the boundaries between these manifestations are unclear, Nock and Favazza [25] have similarly noted it is critical to distinguish between mild, moderate, and severe manifestations of NSSI. Here, the grouping criteria of the number of lifetime NSSI episodes were primarily based on the approach proposed in the K-SADS, in which fewer than four lifetime NSSI episodes were classified as subclinical [18]. Similar approaches to categorizing NSSI severity have been used in other studies [12,26]. The NSSI grouping criteria described in Table 1 has been used in a previous publication [15] in which the Severe and Moderate NSSI groups showed differences in physiological stress response patterns, amygdala and medial prefrontal cortex resting-state functional connectivity, and right amygdala activation to threat as compared to the Mild and No NSSI groups combined. At T1, participants were assigned two NSSI groups, one based on lifetime NSSI engagement (lifetime NSSI group) and another based on NSSI engagement within the past year (past-year NSSI group). At T2 and T3, NSSI group determinations were only based on the NSSI that occurred in the past year.

In addition to measuring suicidal thoughts and behaviors on the SITBI, adolescents completed the self-report Beck Scale for Suicidal Ideation (BSSI), which consists of 19 items that measure the reporter’s suicidal intent. All participants completed the first five items, and those who endorsed items 4 and 5 went on to complete the rest of the items; zeros were substituted in for missing values. The BSSI has been shown to have high internal consistency, moderately high convergent validity, and has demonstrated construct validity through several studies [27].

#### Personality domains

To characterize personality traits in this sample, adolescents completed the Personality Assessment Inventory-Adolescent (PAI-A) [28]. The PAI-A is a 264-item self-report personality assessment consisting of 22 scales, including four validity scales, 11 clinical scales, two interpersonal scales, and five treatment consideration scales. The PAI-A was standardized using both a community and a clinical sample. The average Cronbach’s α for the community and clinical samples were 0.79 and 0.80, respectively, which indicates good internal consistency. The test-retest reliability coefficient for the PAI-A (based on the community sample) was .78, indicating good reliability. Finally, the validity of the PAI-A was verified in relation to other frequently used personality and psychopathology inventories. Analyses focused on the following subscales: Borderline, Depression, Anxiety, Suicide, and Mania. The validity scales, including Inconsistency, Infrequency, Negative Impression, and Positive Impression, were used to determine which data to exclude from analysis. Datasets with validity scores above two standard deviations were omitted. Specifically, if the responses from a participant were too inconsistent (≥78T), infrequent (≥79T), suggestive of a higher degree of psychopathology than what was actually present (≥85T), or indicative of an attempt of the respondent to portray themself without any shortcomings (≥72T), their PAI-A scores were excluded.

#### Childhood trauma experiences

Suicidal and non-suicidal self-injurious behaviors have repeatedly been associated with past histories of trauma and abuse [10,29]. Child Trauma Questionnaire (CTQ) [30] is a self-report instrument covering 28 items that assess the experience of childhood physical and emotional neglect and physical, sexual, and emotional abuse and the self-reported impact of these experiences. The CTQ contains five subscales: physical neglect, emotional neglect, physical abuse, sexual abuse, and emotional abuse, and the scores for each of these range from 5 to 25. The CTQ demonstrates good internal consistency (Cronbach’s α = 0.95), good test-retest reliability, good convergent reliability, and discriminant validity [30].

### Additional Measures to Characterize This Sample

#### Socio-demographic information

At the first visit of each time point, the participant’s parent or caretaker completed a form to report socio-demographic information, including the participant’s date of birth, family income, parental education, occupation, language(s) spoken at home, and race and ethnicity. The Office of Management and Budget standards for race and ethnicity categories were followed to collect the race and ethnicity information (see Supplementary Materials Section S1 for details). On examining all race and ethnicity data collected at T1, we discovered some discrepancies, particularly for situations involving multiple races. These discrepancies were resolved on a case-by-case basis by examining all available information across time points to make a final determination. We acknowledge the limitations of the racial categorization we employed in this study that may have resulted in some of these discrepancies and the need for additional categories and disaggregation of existing ones [31,32]. Youth reports on gender identity were based primarily on the K-SADS-PL [18], which asks for a write-in response to the question, “What is your gender identity?”. Parent-reported gender identity was used when the participant did not provide this information. There are a number of participants that were not asked about their gender identity, given that the earlier versions of the K-SADS-PL used with many of the initial participants did not include questions on gender identity.

### Additional Measures Not Included in This Report

Although not the central focus of this paper, to raise awareness for the readers of the rich clinical, neurocognitive, and neurobiological data that is available from this sample in NDA, the full protocol is described in Supplementary Materials Section S2.

### Statistical Analysis

Descriptive analyses were performed for each measure to characterize the clinical variables at each time point: *Ns*, means, and standard deviations for dimensional scores (e.g., depression severity) and percentages for categorical variables (e.g., rates of psychiatric diagnoses and rates of clinical thresholds for personality domains). *T*-tests and Chi-squared tests were conducted to test for attrition bias. To account for interparticipant score variability and any possible effects of attrition bias on mean scores, linear multilevel modeling was utilized to estimate change in depression severity and suicidal ideation scores over study participation. In addition, from the SITBI, descriptive analyses were conducted on age of onset, motivations, and extent of the following behaviors: suicidal ideation, suicide plan, suicide attempt, NSSI thoughts, and NSSI engagement. Plots were generated showing distributions and trajectories over time using R [33] and SPSS Statistics [34]. One participant was not assigned a past-year NSSI group at T1 as they did not provide sufficient data for group assignment and was not included in analyses involving NSSI groups or related tables and figures.

## RESULTS

### Participants: Sample Description and Retention

Of the 168 participants consented and interviewed at T1, 164 (Mean age = 14.97, SD = 1.20) were considered eligible for this study and were invited for additional visits. 75.61% (124/164) and 57.93% (95/164) of participants who completed the T1 interview returned for interviews at T2 and T3, respectively. Figure 1 shows a consort diagram summarizing the number of participants who completed each stage of the study. Table 2 and Supplementary Materials Section S3 show the demographic characteristics of the sample at T1. Discussion of evaluations for selective retention/attrition is provided subsequently (Attrition Bias).

#### NSSI groups

**Figure 2.**
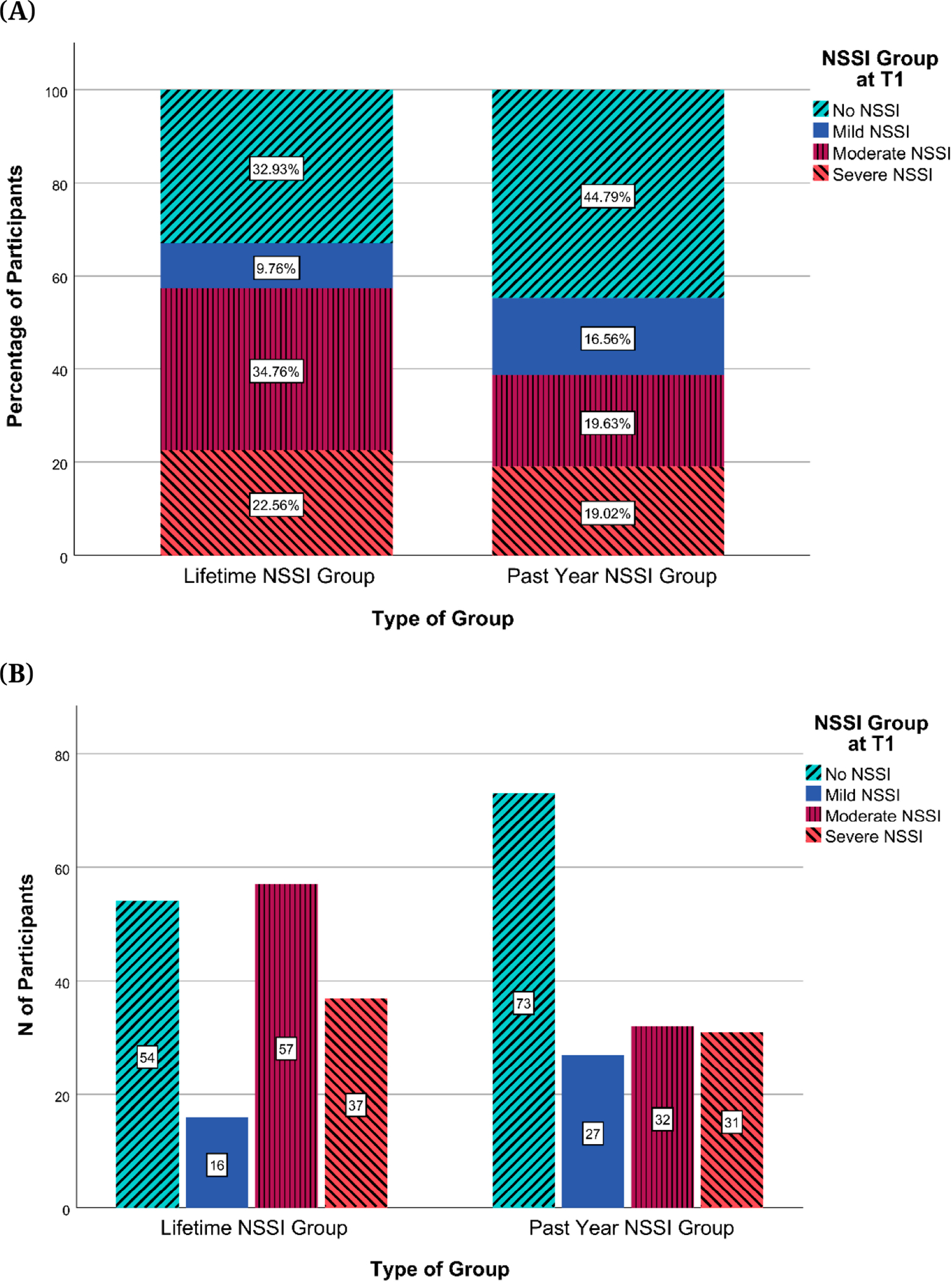
Comparison Between T1 Lifetime and Past-Year NSSI Groups. *Note:* NSSI = Non-Suicidal Self-Injury. (**A**) Percentages of participants by NSSI groups based on lifetime and past year NSSI engagement (**B**) Numbers of participants by NSSI groups based on lifetime and past year NSSI engagement.

**Figure 3.**
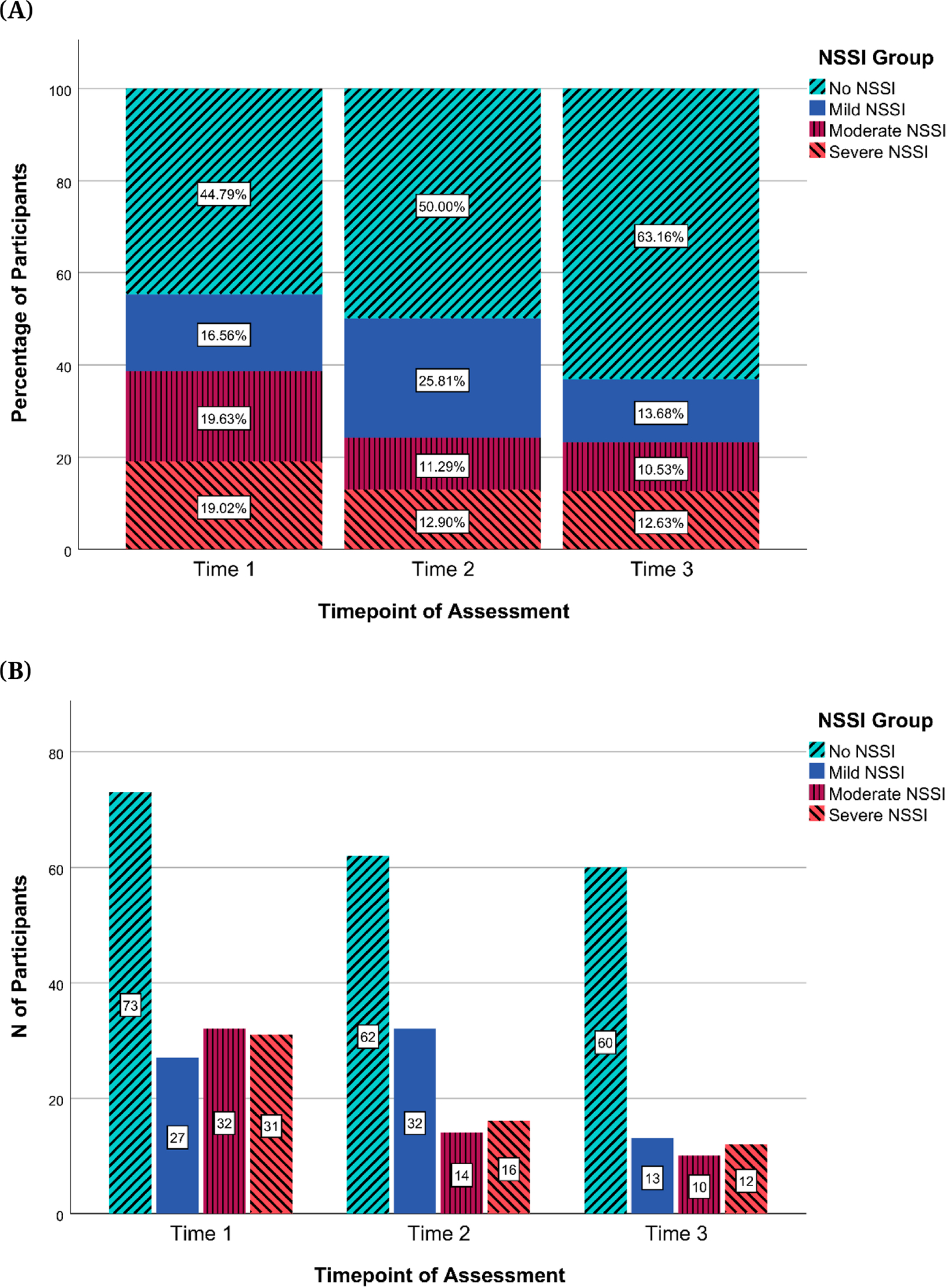
Past-Year NSSI Groups Across Time. *Note:* NSSI = Non-Suicidal Self-Injury. (**A**) Percentages of participants by past-year NSSI groups across time (**B**) Numbers of participants by past-year NSSI groups across time.

**Figure 4.**
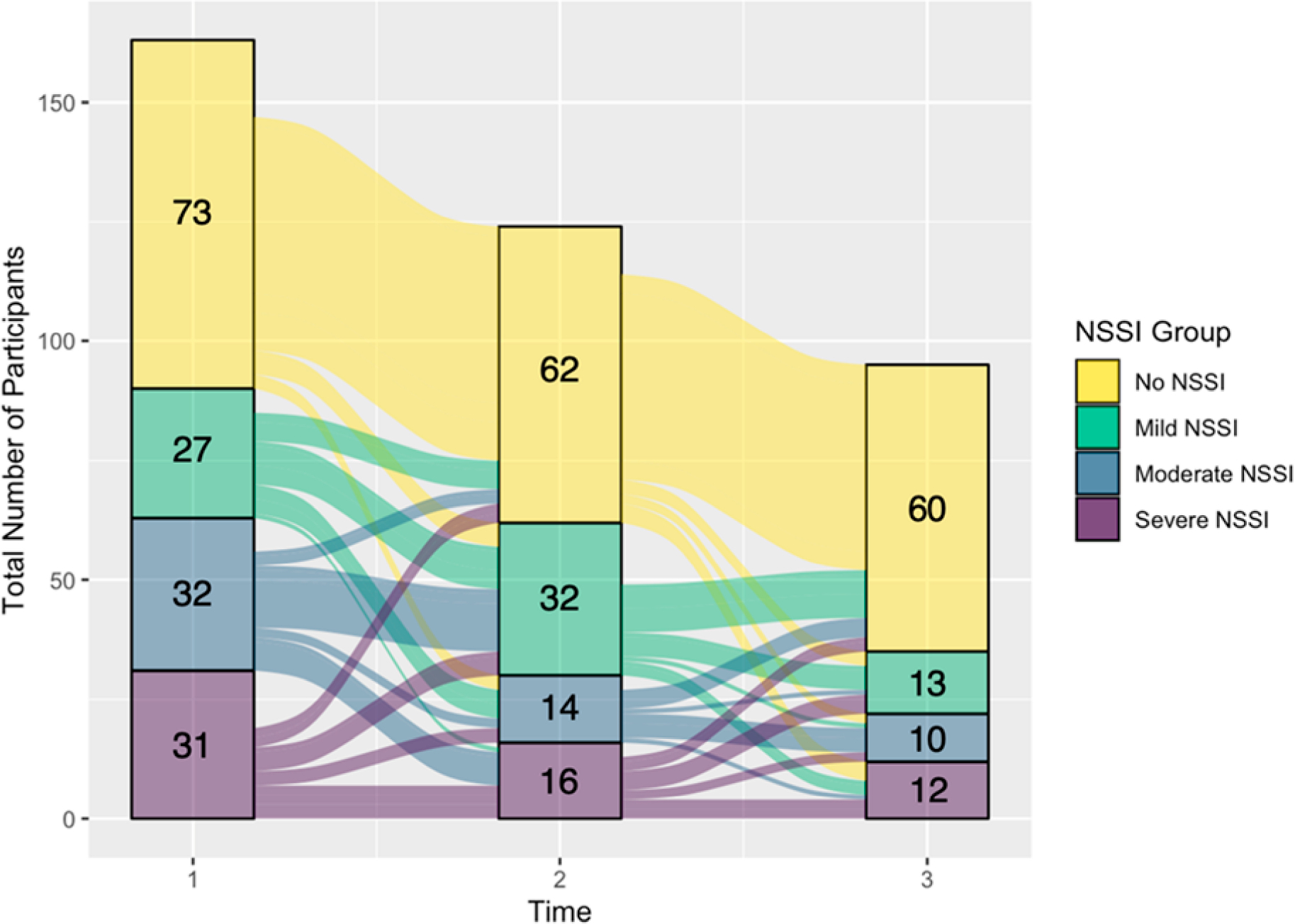
Transitions Between Past-Year NSSI Groups Across T1, T2, and T3. *Note:* NSSI = Non-Suicidal Self-Injury.

Figure 2 shows a comparison between lifetime and past-year NSSI groups assigned at T1, and Figure 3 shows the distribution of past-year NSSI groups at each time point. Figure 2 suggests that Moderate NSSI was the largest group for lifetime NSSI at T1. Figure 3 suggests that for the past-year NSSI group, there was an overrepresentation of participants in the No NSSI group at every time point, with the proportion of participants in this group increasing from T1 to T3. Figure 4 shows the transition of participants between past-year NSSI groups across timepoints: While the majority of participants stayed in the same group between timepoints, some transitioned across groups. More participants shifted from higher to lower severity NSSI groups (27.64% between T1 and T2; 25.26% between T2 and T3) than from lower to higher severity groups (17.88% between T1 and T2; 14.74% between T2 and T3). Additionally, of the 60 participants in the No NSSI group at T3, the majority were already in the No NSSI group at T1 or T2, while the remaining 13.33%, 16.67%, and 6.67% of participants were in the Mild, Moderate, and Severe NSSI groups at T1, respectively, indicating that participants were least likely to have transitioned to the No NSSI group at T3 from the Severe NSSI group. There was no evidence that the participants who transitioned to the No NSSI group by the end of the study from the Mild, Moderate, and Severe NSSI groups were more likely to have been receiving medication management that facilitated this improvement (*p*s > .7, Effect size: Cramer’s V ranged from 0.091 to 0.159).

#### Attrition bias

We wanted to see if there was an attrition bias such that adolescents with certain demographic characteristics or a greater degree of psychopathology were more likely to drop out of the study. In terms of demographic characteristics, there was no evidence of attrition bias based on parent or guardian educational (*p*s > 0.06) or occupational status (*p*s > 0.18). Effect sizes for these comparisons (Cramer’s V) ranged from 0.157 to 0.314. However, there was evidence of family income-based (three of the lowest income categories were combined to form one category due to small group sizes) bias in attrition between T2 and T3 (*p* = 0.041; effect size: Cramer’s V = 0.31) but not between T1 and T2 (*p* = 0.352; effect size: Cramer’s V = 0.188): Participants lost between T2 and T3 were more likely to have been from a lower income group ($40,000–$59,999), whereas participants who continued in the study were more likely to have been from a higher income group ($90,000–$179,999). This difference was initially significant (*p* = 0.012) but did not survive correction for multiple comparisons (corrected *p* = 0.178).

Since this study was conducted over six years and continued through the COVID-19 pandemic, we also assessed whether there was a difference in attrition based on the participants’ enrollment date or if attrition differed before versus after the beginning of the COVID-19 pandemic in Minnesota (15th March 2020). The difference in attrition based on the date of participants’ first appointment of the study was not significant for attrition between T1 and T2 (*p* = 0.984; Cohen’s *d* = 0.004). However, for attrition between T2 and T3, it appeared that participants who returned for T3 appointments were more likely to have joined early in the study (*p* = 0.027; Cohen’s *d* = 0.463). There was no difference in attrition rates between T1 and T2 (*p*s > 0.33) or between T2 and T3 (*p*s > 0.43) before versus after the beginning of the COVID-19 pandemic.

**Figure 5.**
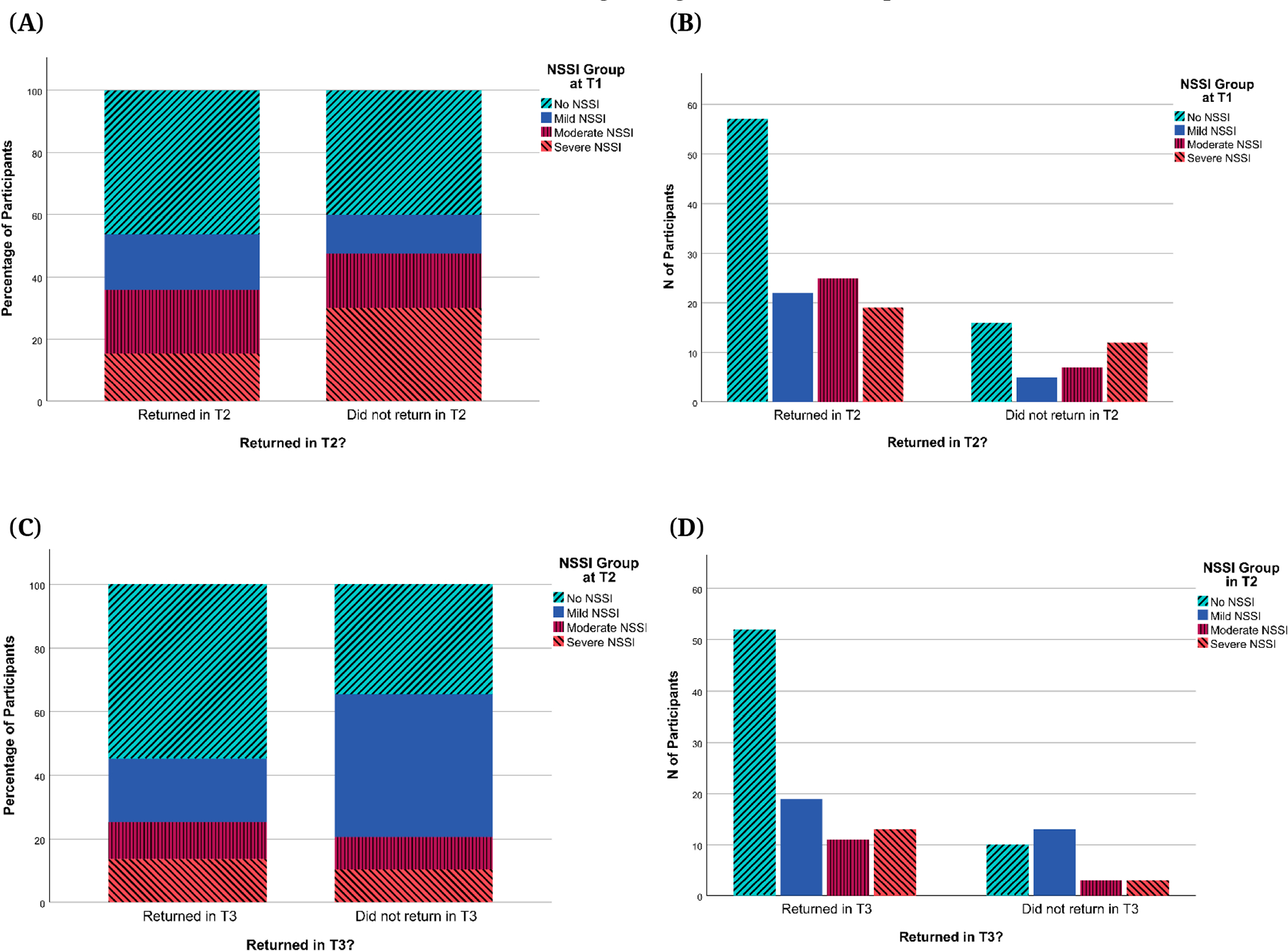
Past-Year NSSI Groups for Returning Participants and Dropouts. *Note:* NSSI = Non-Suicidal Self-Injury. (**A**) Percentages of participants by T1 past-year NSSI groups for participants who returned in T2 and those who did not. (**B**) Numbers of participants by T1 past-year NSSI groups for participants who returned in T2 and those who did not. (**C**) Percentages of participants by T2 past-year NSSI groups for participants who returned in T3 and those who did not. (**D**) Numbers of participants by T2 past-year NSSI groups for participants who returned in T3 and those who did not.

**Figure 6.**
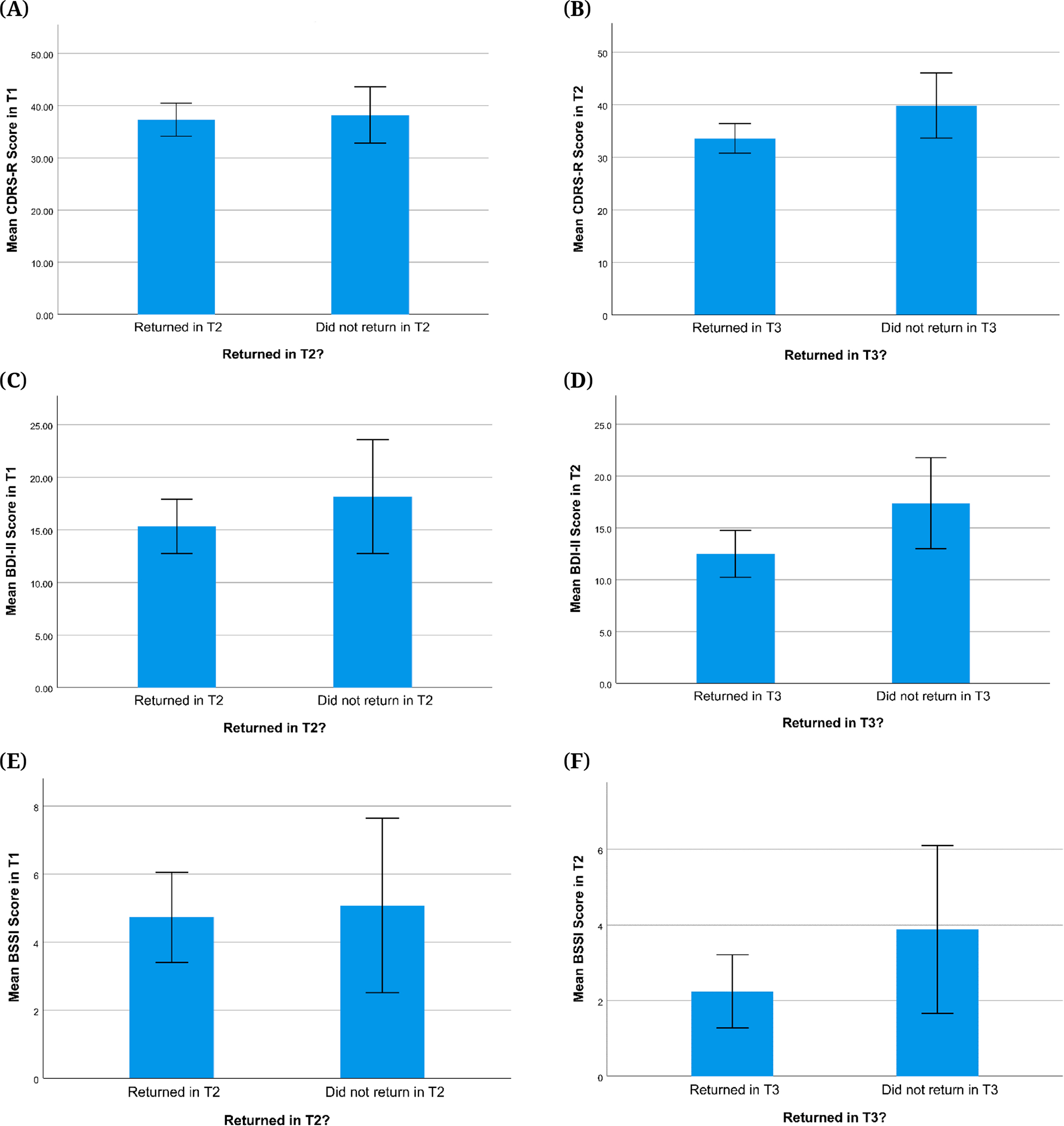
Severity of Depression Symptoms (CDRS-R, BDI-II) and Suicidal Ideation (BSSI) for Returning Participants and Dropouts. *Note.* Error bars are +/− 2 standard error. CDRS-R = Children’s Depression Rating Scale, Revised, BDI-II = Beck Depression Inventory-II, BSSI = Beck Scale for Suicidal Ideation. **(A)** Mean T1 CDRS-R scores for participants who returned in T2 and those who did not. **(B)** Mean T2 CDRS-R scores for participants who returned in T3 and those who did not. **(C)** Mean T1 BDI-II scores for participants who returned in T2 and those who did not. **(D)** Mean T2 BDI-II scores for participants who returned in T3 and those who did not. **(E)** Mean T1 BSSI scores for participants who returned in T2 and those who did not. **(F)** Mean T2 BSSI scores for participants who returned in T3 and those who did not.

**Figure 7.**
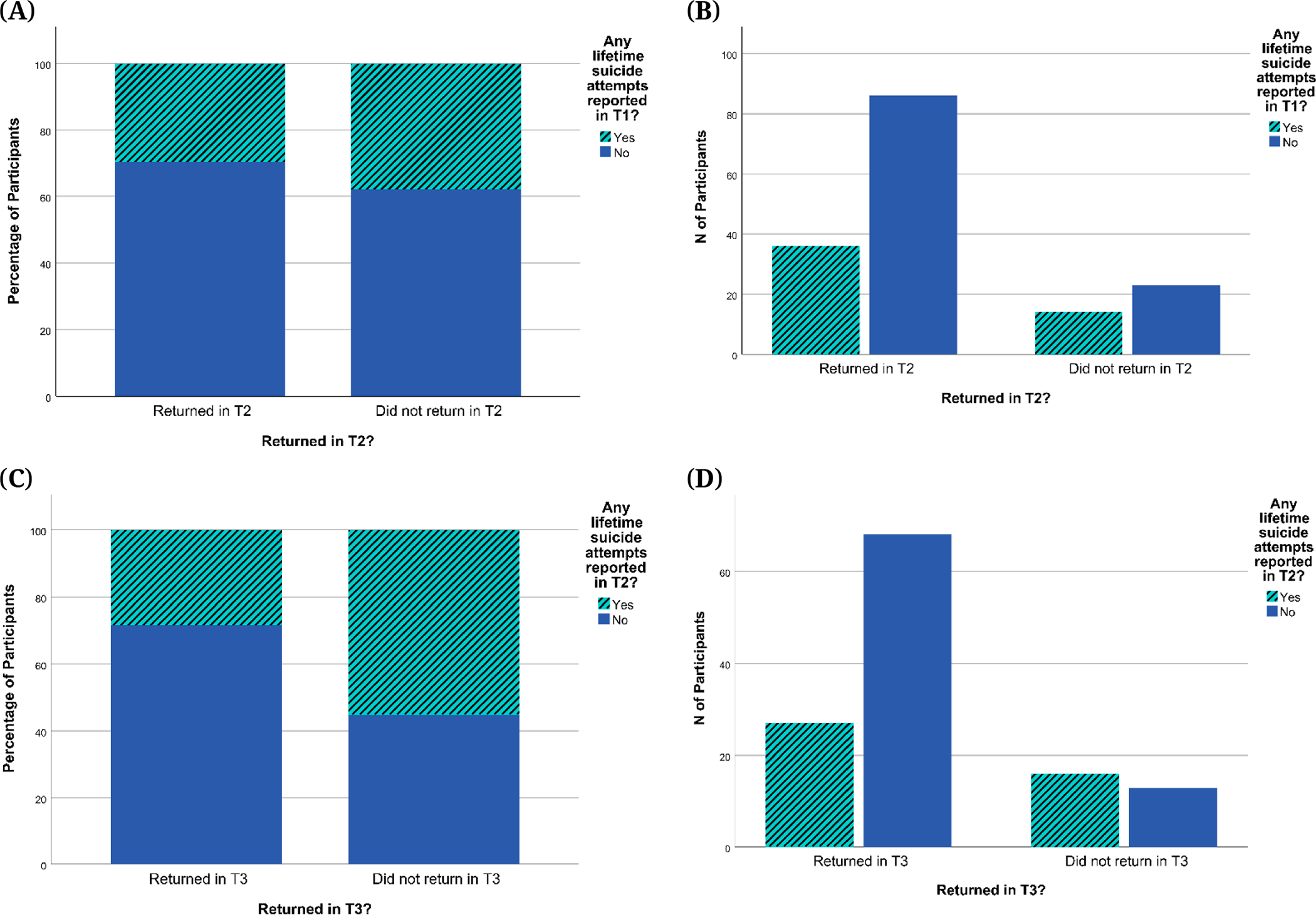
Lifetime Suicide Attempts on the SITBI for Returning Participants and Dropouts. *Note:* SITBI = Self-Injurious Thoughts and Behaviors Interview. (**A**) Percentages of participants reporting any lifetime suicide attempts at T1 for participants who returned in T2 and those who did not; (**B**) Numbers of participants reporting any lifetime suicide attempts at T1 for participants who returned in T2 and those who did not; (**C**) Percentages of participants reporting any lifetime suicide attempts at T2 for participants who returned in T3 and those who did not; (**D**) Numbers of participants reporting any lifetime suicide attempts at T2 for participants who returned in T3 and those who did not.

In terms of psychopathology, when comparing participants who returned for T2 visits versus those who did not, there were no significant differences in NSSI group distribution or T1 scores for depression (CDRS-R, BDI-II), suicidal ideation (BSSI), or childhood trauma (CTQ) (*p*s > 0.232). The same was found when comparing T2 scores for those who did versus did not return for the T3 visit (*p*s > 0.055; see Figures 5 and 6 for NSSI group, depression, and suicidal ideation comparisons and Supplementary Materials Section S4 for childhood trauma comparisons). Effect sizes (Cohen’s d) for tests of biases in attrition between T1 and T2 were small for depression severity (*d* = 0.050 for CDRS-R; *d* = 0.196 for BDI-II) and suicidal ideation (*d* = 0.047 for BSSI). For attrition between T2 and T3, effect sizes were small to medium for depression severity (*d* = 0.433 for CDRS-R ;*d* = 0.450 for BDI-II) and suicidal ideation (*d* = 0.340 for BSSI). While visual inspection of Figures 5 and 6 suggests that returning participants had a higher proportion of those with low depression and suicidal ideation scores and no past-year NSSI history, this difference was not significant. This suggests that there was no evidence of differences between participants who dropped out and those who continued to participate in this study with regard to NSSI history, depression severity, suicidal ideation, and childhood trauma. Similarly, when comparing the number of lifetime suicide attempts reported at T1 for the group of adolescents who did versus did not return for the T2 visit, there were no significant differences (*p* = 0.451) despite a slight overrepresentation of participants with no lifetime suicide attempts in returning participants (see Figure 7). However, we did find that compared to those who completed T2 but not T3, participants who returned for T3 visits were significantly more likely to report no lifetime suicide attempts at T2 (*p* = 0.015; see Figure 7).

### Clinical Assessment

#### Psychiatric diagnoses and treatments

Table 3 shows the rates of psychiatric disorders according to the K-SADS assessments at each visit. The most common diagnoses were Major Depressive Disorder (all time points), followed by Generalized Anxiety Disorder (GAD) at T1, and Attention Deficit Hyperactivity Disorder (ADHD) at T2 and T3 (while ADHD rates remained stable, GAD rates declined, so that ADHD surpassed GAD at the later timepoints in the ranking of diagnosis frequency). Table 4 shows a breakdown of “any mental health diagnosis” and treatments across NSSI groups. Also, our No NSSI group was not intended to be a healthy control. Indeed, rates of mental health diagnoses were greater in the NSSI groups than in the No NSSI group. The number of participants who reported using drugs or alcohol at any point during the study was 13.6%.

#### Severity of depression symptoms and suicidal thoughts

**Figure 8.**
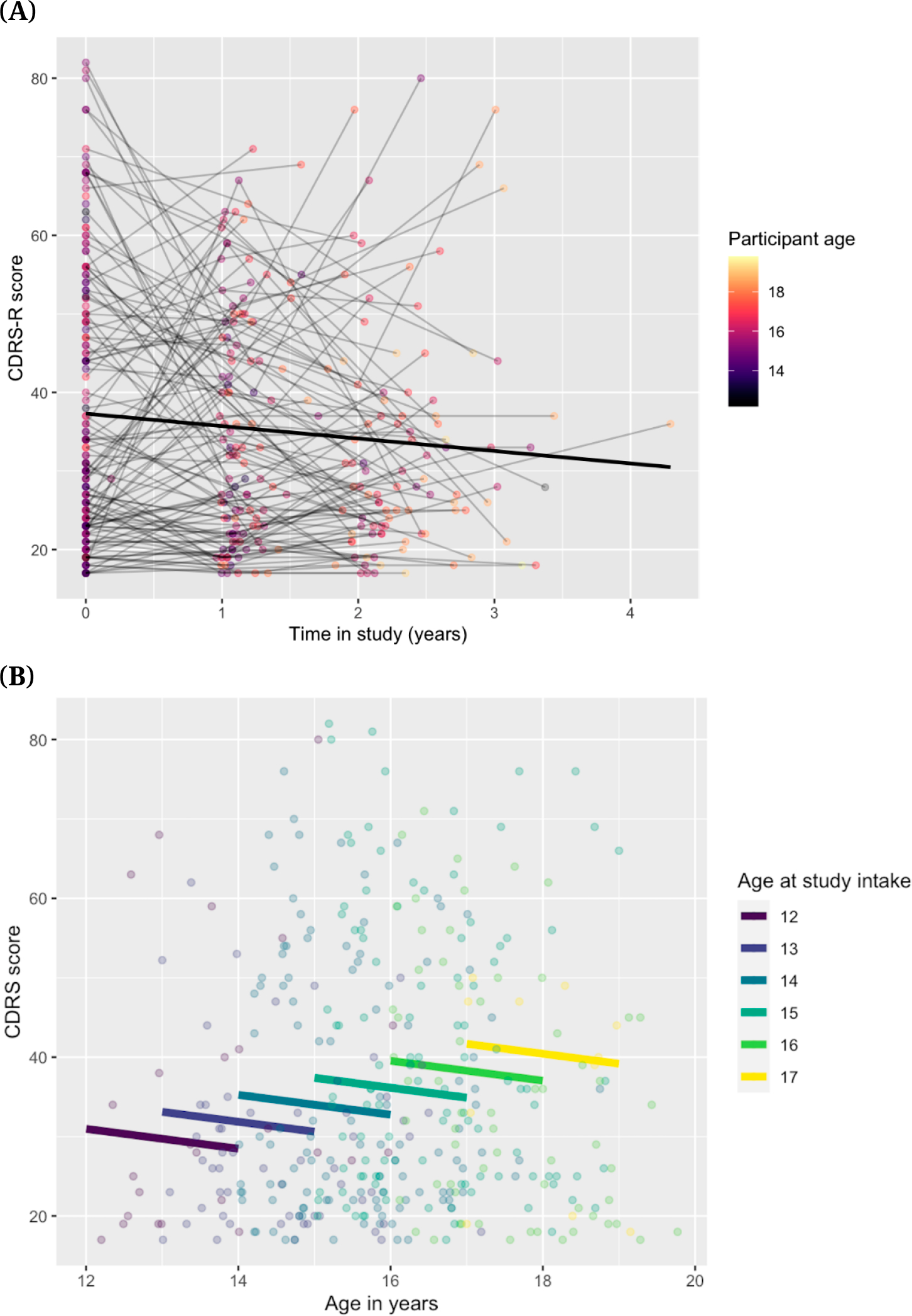
Change Over Time in CDRS-R Scores and Effect of Age at Start. *Note:* CDRS-R = Children’s Depression Rating Scale, Revised. **(A)** Spaghetti plot showing change over time in CDRS-R scores **(B)** Effect of age-at-start on CDRS-R Scores.

**Figure 9.**
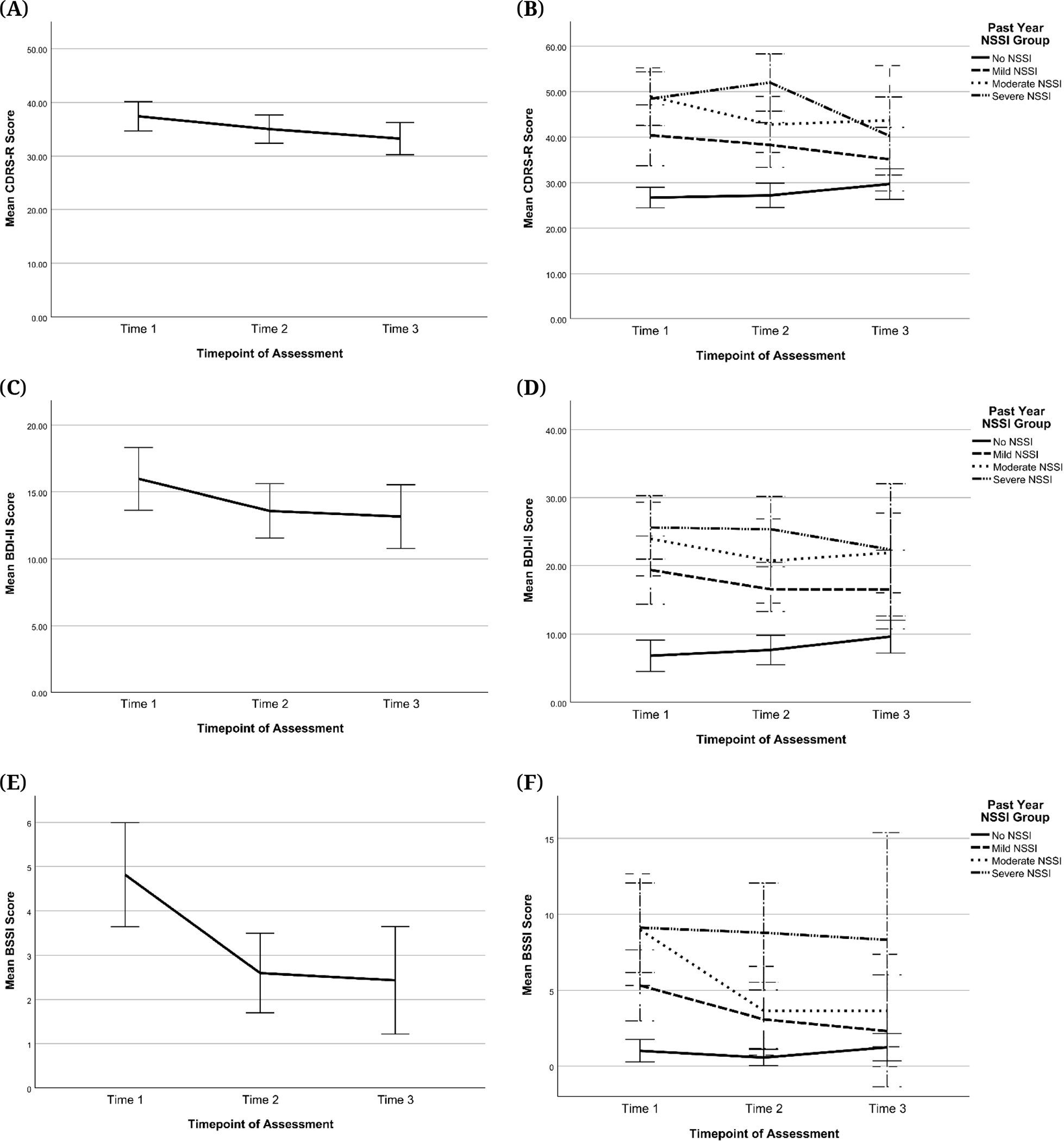
Changes in CDRS-R, BDI-II, and BSSI Scores Across Time for All Participants (left side) and by Past-Year NSSI Group (right side). *Note:* Scores at T1, T2, and T3 were categorized by past-year NSSI groups assigned at the respective timepoints. Error bars are +/− 2 standard error. CDRS-R = Children’s Depression Rating Scale, Revised, BDI-II = Beck Depression Inventory-II, BSSI = Beck Scale for Suicidal Ideation, NSSI = Non-Suicidal Self-Injury. **(A)** Change in CDRS-R sample mean across time **(B)** Change in CDRS-R scores across time by past-year NSSI group **(C)** Change in BDI-II sample mean across time **(D)** Change in BDI-II scores across time by past-year NSSI group **(E)** Change in BSSI sample mean across time **(F)** Change in BSSI scores across time by past-year NSSI group.

Table 5 shows the Ns, means, and standard deviations for CDRS-R, BDI-II, and BSSI scores at each time point. Overall, there was a pattern of decreasing severity for depression and suicidal thoughts in this sample over time (see Figures 8 and 9). As shown in Figure 8A, for CDRS-R scores, this average trend was observed in the context of substantial variability in the sample with respect to change over time. Figure 8A also highlights the variability in actual time elapsed between visits, which was most variable for the T3 visit. As shown in Figure 8B, there was an effect of age in which adolescents who started the study at an older age tended to have higher baseline depression scores, followed by similar decreases over time across age-at-baseline sub-groups. Similar patterns were observed for BDI-II and BSSI scores (see Supplementary Materials Section S5). Figure 9 also shows changes in depression severity and suicidal ideation scores over time by NSSI groups. Overall, participants with no history of NSSI in the past year (No NSSI group) appear to have the lowest CDRS-R, BDI-II, and BSSI scores at any time, whereas the Severe and Moderate NSSI groups appear to have the highest. While statistical tests were not conducted to test the significance of these differences, visual inspection of the graphs suggests that a more severe NSSI history corresponds with more severe depression severity and suicidal ideation. There also appears to be a difference in overall changes in these scores by NSSI group, such that scores seem to increase over time for the No NSSI group but decrease for the three NSSI groups.

#### Self-injurious thoughts and behaviors

164 participants completed the SITBI at T1; 124 at T2; 95 at T3.

##### Age of onset.

**Figure 10.**
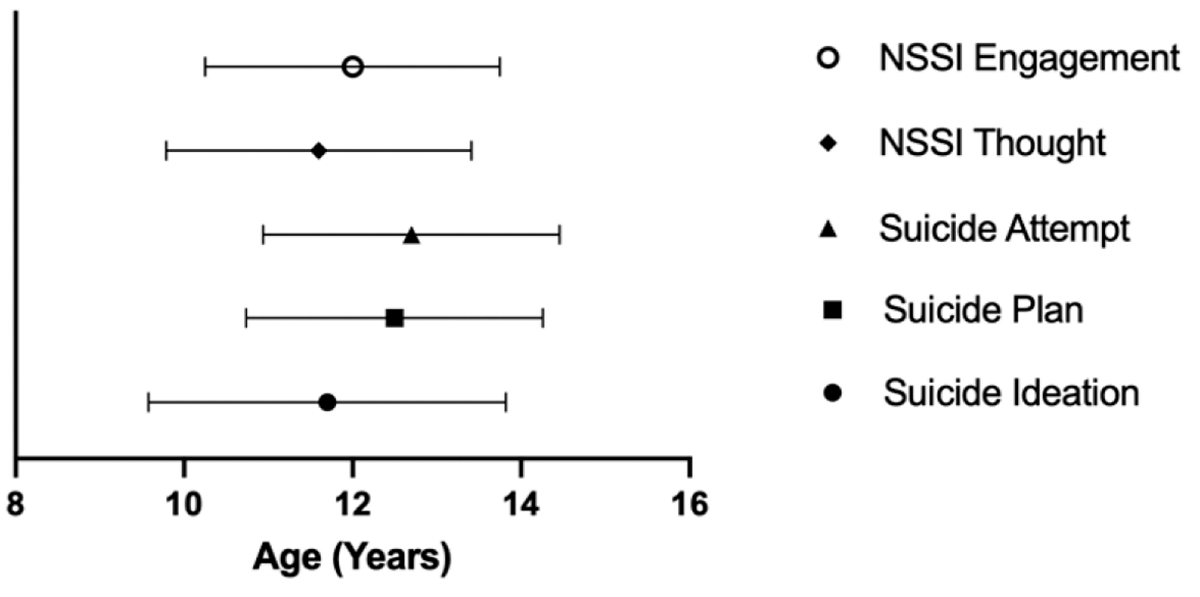
Age of First Suicidal Ideation, Suicide Plan, Suicide Attempt, NSSI Thoughts, and NSSI Engagement as Reported at Time 1. *Note:* NSSI = Non-Suicidal Self-Injury.

Information on age of onset was collected through the SITBI at T1, T2, and T3. However, there were discrepancies in ages of onset reported at the different timepoints. This was not surprising since memory of self-injurious thoughts and behaviors are known to be biased [35]. In our sample, for example, of the 83 participants who provided an age for their first NSSI engagement at more than one timepoints, 27 participants (32.53%) reported the same age at all timepoints for which their data were collected, whereas the other 56 participants (67.47%) provided discrepant data: age of first NSSI engagement they provided was different at least one of the timepoints. Despite these discrepancies, the mean age of onset for NSSI engagement stayed relatively stable across timepoints: mean (SD) reported at T1 = 12.0 (1.75), T2 = 12.0 (2.19), and T3 = 12.2 (2.0). Hence, the following age of onset information only includes T1 data. As shown in Figure 10, the mean age for the onset of first NSSI thoughts was 11.6 (SD = 1.81) years (65% of total participants at T1); the mean age of first NSSI engagement was 12.0 (SD = 1.75) years (65% of total participants at T1). Of the 109 participants who reported NSSI thoughts, all of them (100%) reported going on to NSSI engagement. The mean age of onset for suicidal ideation (SI) was 11.7 (SD = 2.12) years (65% of total participants T1 had SI), and the mean age of first suicide plan was 12.5 (SD = 1.76) years (37% of total participants T1), of which 62/70 (88.5%) actually went on to an attempt. The mean age for first suicide attempt was 12.6 (SD = 1.77) years.

##### Suicide attempts.

At T1, 54/164 (32.9%) of participants reported a lifetime suicide attempt, of which 34/54 (62.9%) were in the past year. At T2, 43/124 (34.6%) of participants reported a lifetime suicide attempt, of which 15/43 (34.9%) were in the past year. At T3, 29/95 (30.5%) of participants reported a lifetime suicide attempt, of which 9/29 (31.0%) were in the past year. Therefore, the highest number of reported lifetime suicide attempts in this study was at T1, and the number of attempts reported decreased at T2 and T3.

**Figure 11.**
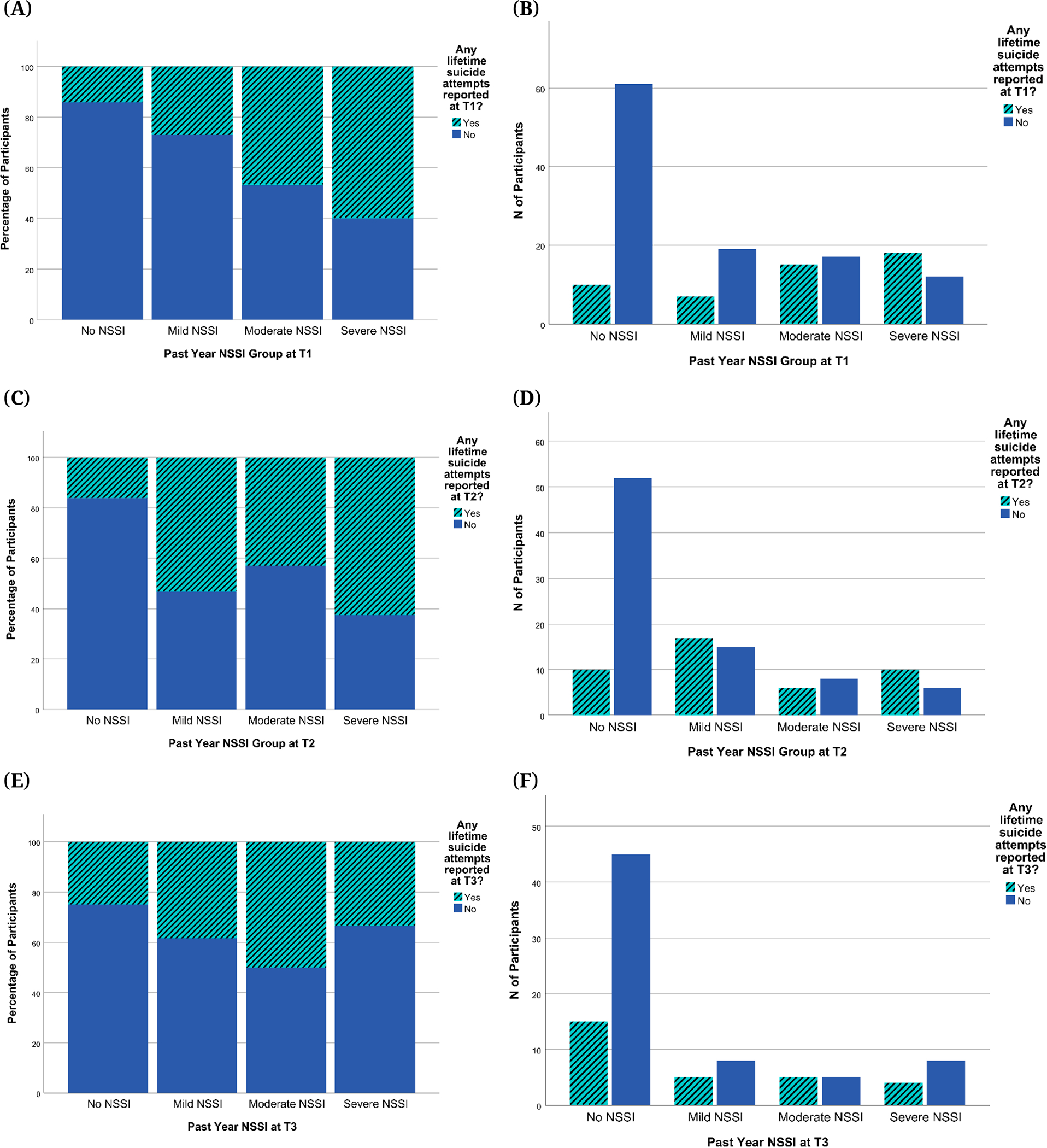
Lifetime Suicide Attempts by Past-Year NSSI Group. *Note:* NSSI = Non-Suicidal Self-Injury. (**A**) Percentages of participants reporting lifetime suicide attempts at T1 by past-year NSSI groups assigned at T1 (**B**) Numbers of participants reporting lifetime suicide attempts at T1 by past-year NSSI groups assigned at T1 (**C**) Percentages of participants reporting lifetime suicide attempts at T2 by past-year NSSI groups assigned at T2 (**D**) Numbers of participants reporting lifetime suicide attempts at T2 by past-year NSSI groups assigned at T2 (**E**) Percentages of participants reporting lifetime suicide attempts at T3 by past-year NSSI group assigned at T3 (**F**) Numbers of participants reporting lifetime suicide attempts at T3 by past-year NSSI groups assigned at T3.

Figure 11 shows the distribution of lifetime suicide attempt reports by NSSI group at each timepoint. Figure 11 suggests that at every time point, the percentages of participants reporting a lifetime suicide attempt were larger in the Mild, Moderate, and Severe NSSI groups than in the No NSSI group. This implies that participants reporting a lifetime suicide attempt at any time were more likely to have engaged in NSSI during the past year than not. Within the NSSI groups, percentages of participants reporting a lifetime suicide attempt appeared to increase with increasing NSSI severity at T1 but not at T2 or 3.

**Figure 12.**
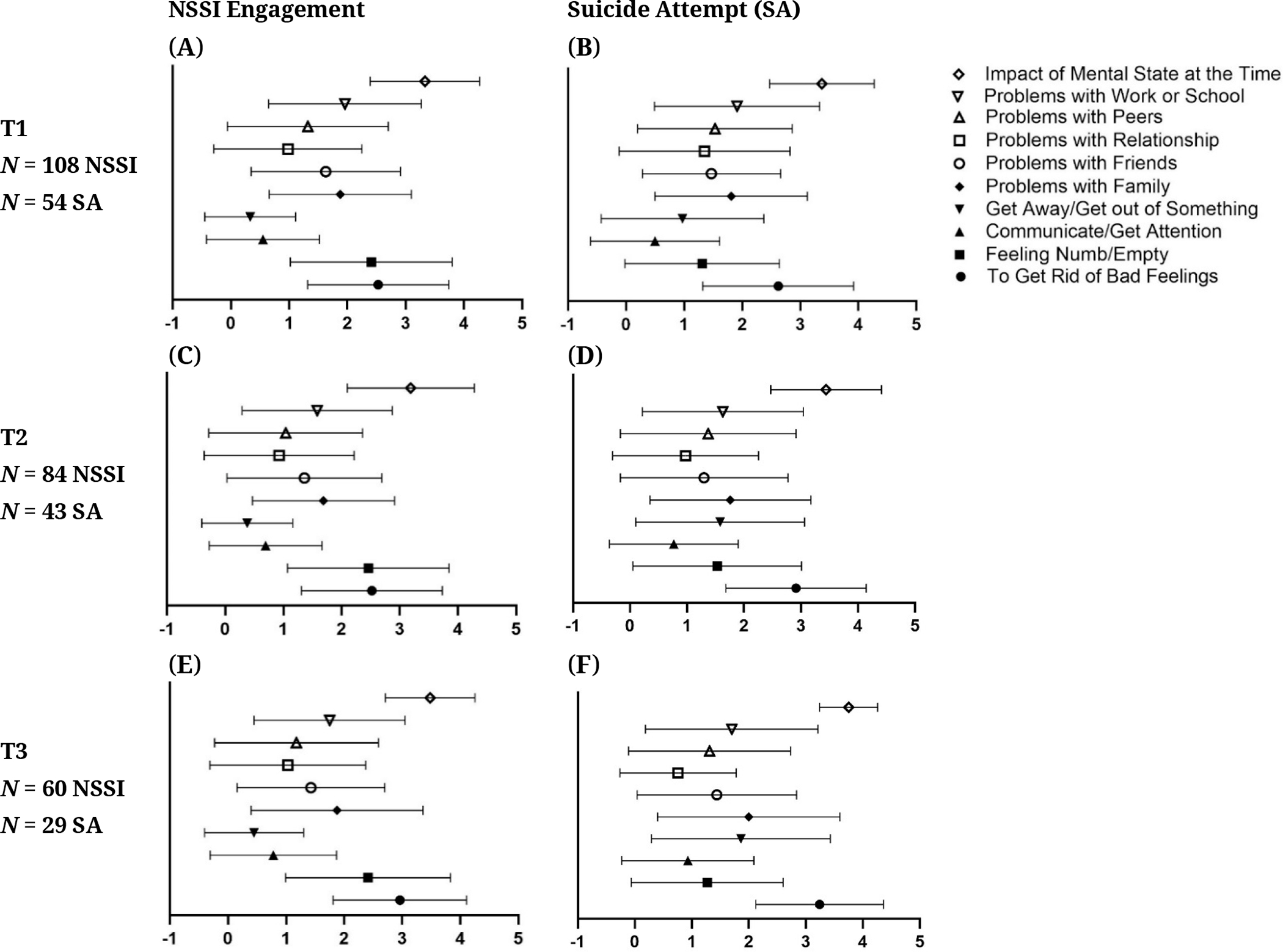
Reported Reasons for NSSI and SA Across Time. *Note:* NSSI = Non-Suicidal Self-Injury, SA = Suicide Attempt.

Motivation for self-harm was assessed with the SITBI both quantitatively and qualitatively. For each class of suicidal thoughts and behaviors, participants rated the extent to which they agreed that their motivation fit with a list of options on a scale ranging from 0 (low/little) to 4 (very much/severe). Means and SDs for these endorsements for NSSI and suicide attempts at T1, T2, and T3 are shown in Figure 12. The most common reasons for NSSI were “Impact of Mental State at the Time”, “To Get Rid Of Bad Feelings”, and “Feeling Numb/Empty”. The most common reasons for suicide attempts were “Impact of Mental State at the Time”, “To Get Rid Of Bad Feelings”, “Problems with Work or School”, and “Problems with Family”. Qualitative reasons (reasons provided by participants in their own words) for SI, Suicide Plan, Suicide Attempt, NSSI thought, and NSSI engagement as described by participants at T1 can be found in Supplementary Materials Section S6. The most common free-form answers that participants reported for SI, Suicide Plan and Suicide Attempt were “Don’t Know”, “Feeling Sad/Depressed/Bad Feelings”, “Low Self-Esteem/Self-Hatred/Unhappiness with Body”, and “Hopelessness/Life has no Meaning/No Future”. The most common answers for NSSI thought and engagement included those mentioned above, and “Feel Something/Address Numbness”.

#### Personality domains: PAI-A

114 participants completed the PAI-A at T1, 78 at T2, and 62 at T3. 26 participants were excluded due to having invalid scores on at least one of the validity scales. Table 6 shows the distribution of invalid PAI-A responses in terms of the type of invalidity and the past-year NSSI group of the responder. As shown in the table, participants who reported more severe NSSI engagement in the previous year were more likely to have PAI-A validity scale scores in the “Caution” range. Chi-square tests indicated that this difference was statistically significant (*p* = 0.029), and multilevel logistic regression accounting for intra-individual consistency in response style confirmed this as well. However, it is important to note that most of these validity flags were due to inconsistent response style and endorsing infrequent items. Given the length of the PAI-A, it is possible that participants with more severe NSSI and more overall psychopathology may have shown more variation in their responses on this particular measure and/or endorsed items with higher item response theory thresholds. These validity flags were not used to exclude these participants’ responses on other questionnaires, as other measures were shorter in length and less likely to suffer from inconsistent response issues and occasionally contain their own validity indicators of inconsistent responses.

**Figure 13.**
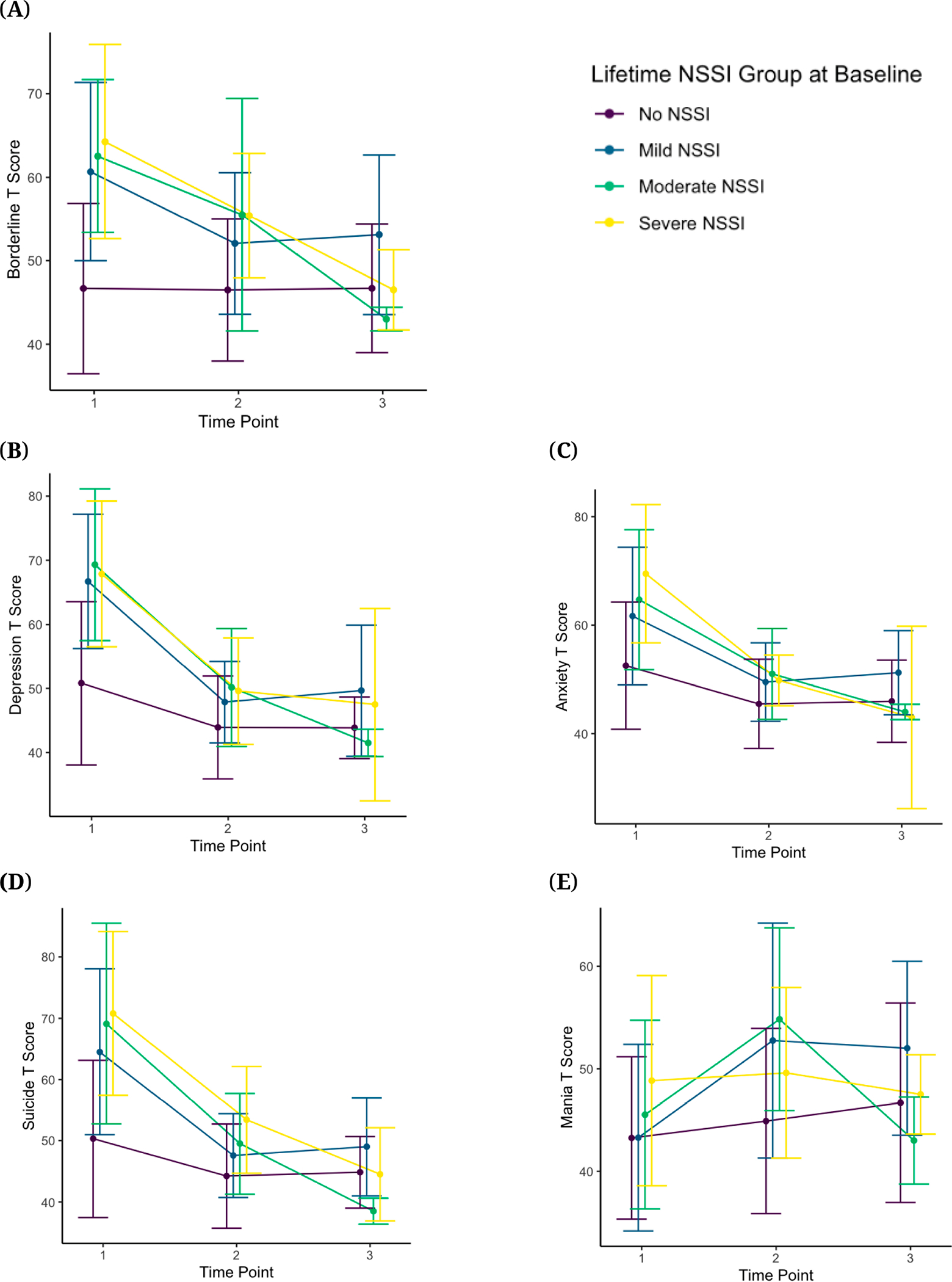
Changes in PAI-A Scores Across Time by NSSI Group. *Note:* Error bars represent 1 Standard Deviation. PAI-A = Personality Assessment Inventory–Adolescent, NSSI = Non-Suicidal Self-Injury. (**A**) PAI-A borderline t-scores across time by NSSI group; (**B**) PAI-A depression t-scores across time by NSSI group; (**C**) PAI-A anxiety t-scores across time by NSSI group; (**D**) PAI-A suicide t-scores across time by NSSI group; (**E**) PAI-A mania t-scores across time by NSSI group.

Table 7 shows means and standard deviations for several key scales (Borderline, Depression, Anxiety, Suicide, and Mania) by NSSI group, as well as the rates of clinically significant scores for each group at each time point. As shown in Table 7, out of all the longitudinal PAI-A datasets (after excluding one participant who had no NSSI group), 23 (from 17 participants) were at or above threshold for severe clinical significance (Borderline = 3, Depression = 5, Anxiety = 6, Suicide = 6, Mania = 3). Of these 23 datasets, 14 datasets had PAI-A data at T1 only, six had data at follow-up but still scored at or above this threshold on these subscales at T1, and three scored at or above threshold on these scales at T2. No participant scored at or above the clinical threshold at a T3 visit. Five participants met clinical threshold on two subscales, and one participant met clinical threshold on three subscales, all of which occurred at T1. Thus, after removing these additional datasets, nine participants with clinically significant scores on a subscale dropped out after T1, six participants dropped after T2, and one participant completed all three visits (additionally, one participant completed the PAI-A at T1 and T3, but not at T2). Figure 13 shows the trends over time by baseline NSSI group. As shown, overall, there is a decrease in severity across the PAI-A scales highlighted here, with the exception of Mania scores showing an increase over time, especially for the adolescents who were included in the Mild and Moderate NSSI groups at baseline.

#### Childhood trauma experiences

**Figure 14.**
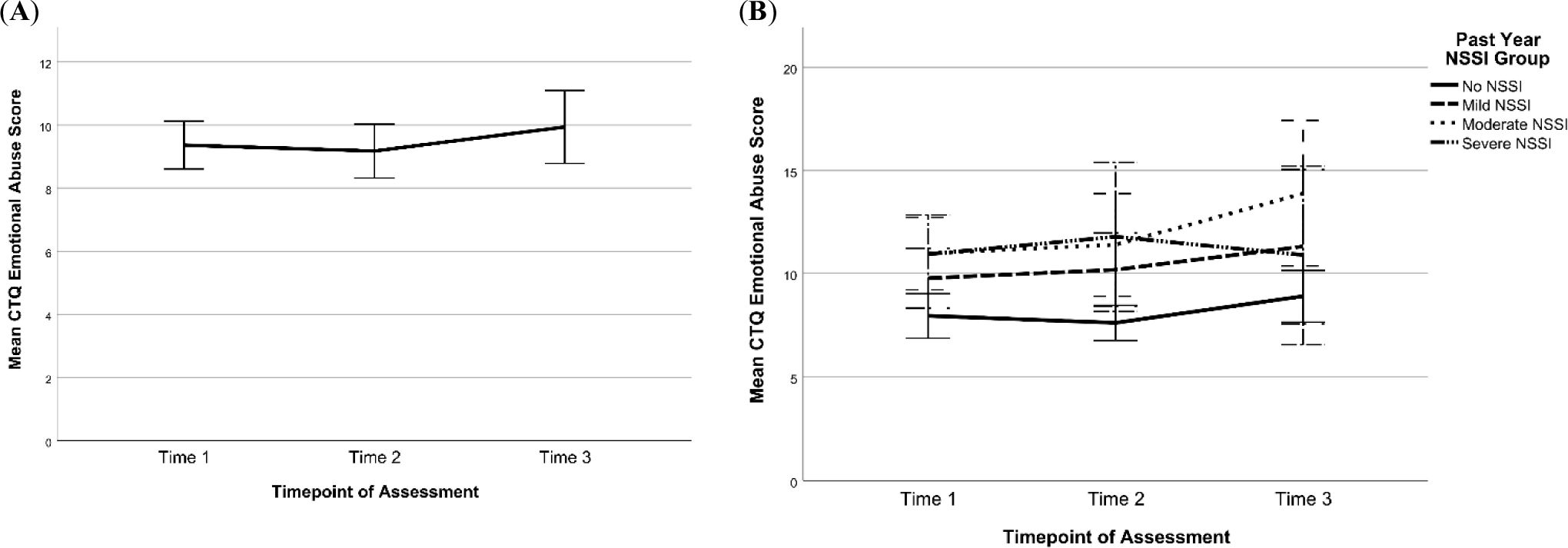

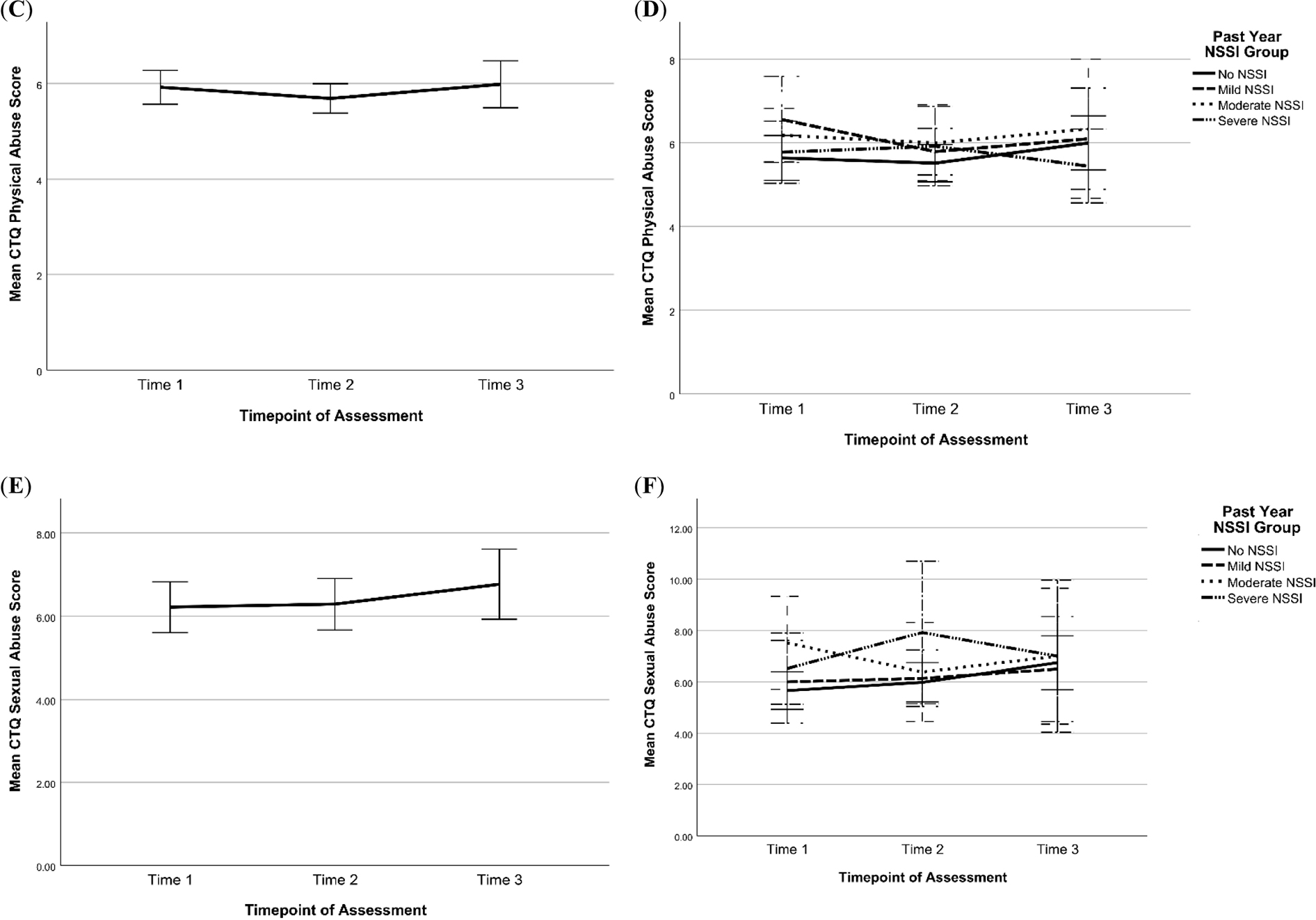
Changes in Emotional, Physical, and Sexual Abuse Scores on the CTQ Across Time for All Participants (left side) and by Past-Year NSSI Group (right side). *Note*: Scores at T1, T2, and T3 were categorized by past-year NSSI groups assigned at the respective timepoints. Error bars are +/− 2 standard error. CTQ = Child Trauma Questionnaire, NSSI = Non-Suicidal Self-Injury. (**A**) Change in CTQ emotional abuse sample mean across time; (**B**) Change in CTQ emotional abuse scores across time by past-year NSSI group; (**C**) Change in CTQ physical abuse sample mean across time; (**D**) Change in CTQ physical abuse scores across time by past-year NSSI group; (**E**) Change in CTQ sexual abuse sample mean across time; (**F**) Change in CTQ sexual abuse scores across time by past-year NSSI group.

**Figure 15.**
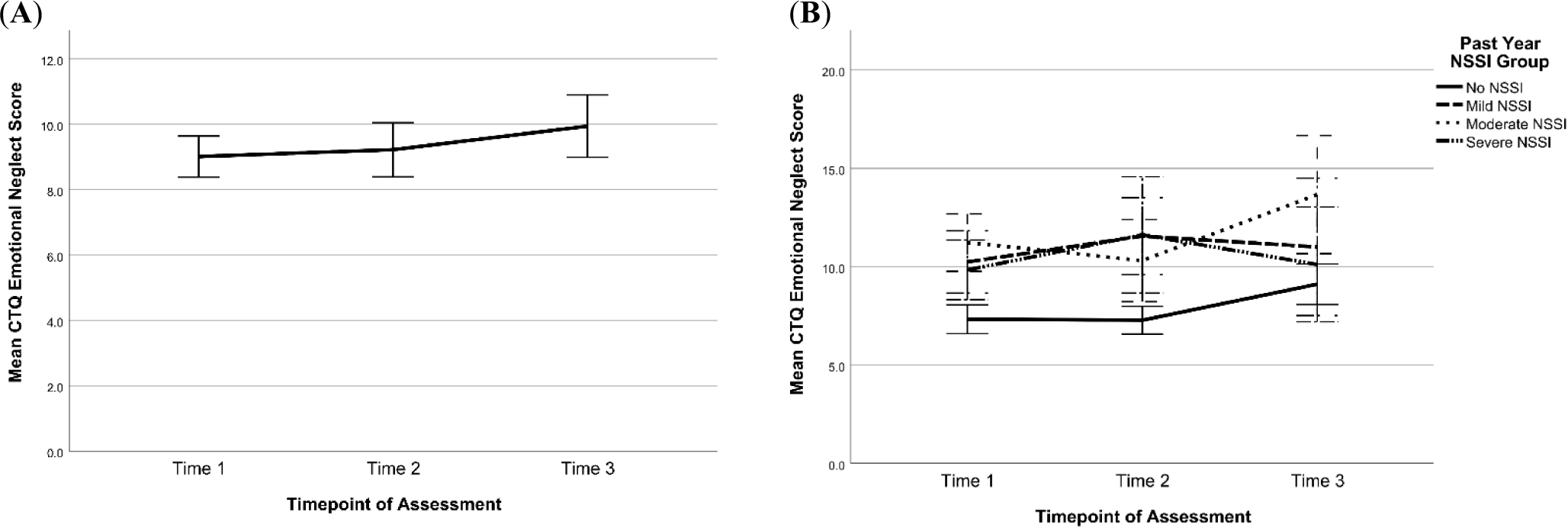

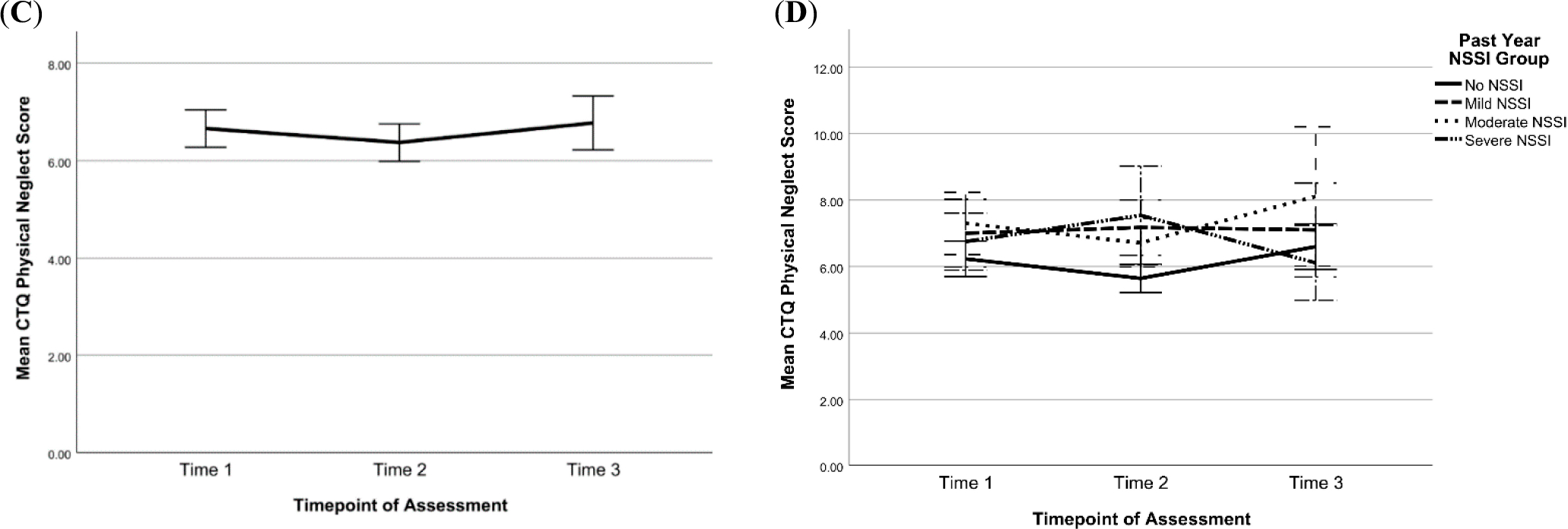
Changes in Emotional and Physical neglect Scores on the CTQ Across Time for All Participants (left side) and by Past-Year NSSI Group (right side). *Note:* Scores at T1, T2, and T3 were categorized by past-year NSSI groups assigned at the respective timepoints. Error bars are +/− 2 standard error. CTQ = Child Trauma Questionnaire, NSSI = Non-Suicidal Self-Injury. (**A**) Change in CTQ emotional neglect sample mean across time (**B**) Change in CTQ emotional neglect scores across time by past-year NSSI group (**C**) Change in CTQ physical neglect sample mean across time (**D**) Change in CTQ physical neglect scores across time by past-year NSSI group.

Respectively, 147, 114, and 83 participants completed the CTQ at T1, T2, and T3 based on lifetime reports. Table 8 summarizes the means and SDs for scores at each time point for the domains of past physical abuse, emotional abuse, sexual abuse, emotional neglect, and physical neglect. Overall, mean scores were similar across time points, with minor increases in reports of past emotional and sexual abuse and emotional neglect (see Figures 14 and 15). Figures 14 and 15 also show changes in CTQ subscales over time by NSSI Groups. Visual inspection of the graphs suggests that across all time points, emotional abuse and neglect scores were lowest for participants with no NSSI history in the past year (No NSSI group). For the remaining CTQ subscales, with respect to NSSI groups, no other consistent patterns emerged.

## DISCUSSION

We provide longitudinal clinical data on a group of adolescents who were assigned female sex at birth and had a range of histories of NSSI ranging from none to severe at baseline. Key strengths of the study include the longitudinal study design and multimodal approach, which has resulted in a rich, publicly available dataset that can be used to address many different kinds of questions to advance our understanding of adolescent NSSI and its neurodevelopmental correlates. The breadth of longitudinal data collected from this sample of adolescents enabled us to capture the progression of NSSI during this critical developmental period and its temporal relationships with commonly implicated risk and protective factors. Here we have provided a detailed description of these adolescents’ clinical presentation and trajectories over time with respect to depression, NSSI, STBs, personality, and childhood trauma experiences.

### Clinical Assessment

One key observation in this dataset is that, overall, decreases in the severity of psychopathology were seen over time. This may reflect a combination of multiple processes. First, this may be capturing a true natural course. It may be that psychopathology at study entry tends to be more severe and that over time, these issues become less severe, perhaps due to interventions (e.g., psychotherapy), developmental processes, or natural course of the problems. Another factor may be a potential bias in attrition patterns, in which participants with more severe and persistent psychopathology may be more likely to drop out. Of note, much of our clinical data (e.g., CDRS-R, BDI-II, and BSSI) did not show evidence of biased attrition. However, 14 of the 17 participants who scored above threshold on one or more of the PAI-A clinical subscales scored above threshold at T1, with over half of these participants dropping out after their first visit. These elevated scores indicate the high likelihood of a diagnosis of borderline personality disorder (three participants) and major depressive disorder (five participants). Additionally, at the time of these reports, six participants likely suffered from significant clinical presentations of anxiety, six struggled with severe and active suicidality, and three experienced symptoms typically associated with a diagnosis of mania. Potentially, these individuals’ symptomatology (and related life circumstances) may have made it difficult to remain in the study and complete all of the tasks required of them.

### NSSI Group Differences

An important goal of the BRIDGES study was to explore differences in longitudinal trajectories between adolescents with and without NSSI histories and across a range of NSSI severity. Reassessing the NSSI history of participants at every time point allowed us to record possible changes in NSSI engagement across adolescence. While we aimed to recruit equal numbers of participants in each NSSI group, there was an increase in size of the group of adolescents who were not engaging in NSSI from T1 to T3, possibly because this group was slightly more likely to return for subsequent visits than the other groups, even though group differences in attrition were found to be non-significant. NSSI is frequently described as a maladaptive way of managing intense distress [36], so it may be that participants who were not experiencing as much distress in their lives were less likely to engage in NSSI and more likely to return for subsequent visits. While our goal in the current paper was to describe our data rather than conduct statistical tests of differences in psychopathology across NSSI groups, visual inspections of the data suggest an association between NSSI history and depression, suicidal ideation, suicide attempt, emotional abuse and neglect. These observations are consistent with research suggesting elevated depression, suicidal ideation, childhood trauma, and risk of future suicide attempts in adolescents engaging in NSSI [1,8–10].

### Suicide Attempts

An important feature of the BRIDGES study is the detailed longitudinal data on suicide risk in a sample that is enriched for increased risk. In our sample, following the baseline assessment, there were 24 new suicide attempts reported during study follow-up. This is most likely an underestimate in the sample due to the loss of high-risk participants to follow-up. A systematic review of clinical studies assessing self-harm in adolescents showed that, on average, 33% of participants in the nontreatment group reported some form of self-harm, although this varies widely based on the study [37]. Further research using this dataset may be useful in identifying risk factors for future suicide attempts, which could potentially inform suicide prevention strategies.

### Responding to Suicide Risk

With safety as the utmost priority, it is important for research studies involving adolescents at elevated risk for suicide to consider how to support these youth by making the most appropriate referral strategies. Some studies have shown that providing care to adolescents at high risk for suicide can be a challenge, with frequent instances of noncompliance or dropping out of treatment [38]. This may reflect an inherent instability in the population. For example, a study testing a method of periodic phone calls to stay in touch with high-risk adolescents over a one-year follow-up period showed that the adolescents who were unreachable at one year had higher rates of school dropout and relocations [39]. Growing evidence supports the use of dialectical behavioral therapy (DBT) in adolescents with self-injury and suicide risk. To date, one randomized control trial of adolescents at high risk for suicide demonstrated strong evidence for DBT as an effective treatment for decreasing repeated suicide attempts and self-harm [40]. Additional research is needed to identify effective treatment options that are accessible, acceptable, and engaging for high-risk adolescents and their families.

### Age of Onset Clinical Considerations

A clinically important observation from this dataset is regarding the age of onset. The mean age for the onset of first NSSI thoughts was 11.6 years, and the mean age of first NSSI engagement was 12.0 years. The mean age of onset for SI was 11.7 years, the mean age of first suicide plan was 12.5 years, with 88.5% going on to make an attempt, and the mean age for first suicide attempt was 12.6 years. This data enhances knowledge for clinical providers who work with adolescents to be more fully informed about when these concerns may begin. It supports the regular implementation of thorough suicide and NSSI assessment (thoughts and behaviors) in late childhood and early adolescence for those who may be at risk. This data can also inform prevention efforts to begin early enough in development to have the intended impact. Depression prevention programs, for example, target ages 11–15 which is considered the time when symptoms are present but not yet a diagnosable disorder [41]. Adding to prior knowledge about the overlap between depression, NSSI, and STBs [11], major depression was the most common diagnosis in this sample. Therefore, the data from this study could help inform the understanding of the course of depression illness and ways to optimize prevention and intervention efforts targeting NSSI and suicidality.

### Attrition

The higher-than-expected attrition rates in this study may be due to several factors. First, the study focused on a population characterized by significant stress and distress. Our sample included adolescents with challenging family situations and recurring psychiatric emergencies requiring recurrent hospitalizations and, sometimes, placements out of the home. Although we were not always able to ascertain information from families regarding their reasons for attrition, conceivably, such challenging circumstances could have led some of the adolescents to discontinue participation. Second, the study protocol included a large number of clinical assessments (extensive interviews, many questionnaires about symptoms and experiences) which are time-consuming and, at times, emotionally taxing. The protocol also included an experimental stress paradigm and a lengthy MRI session that included many different scans, one of which included negatively valanced visual stimuli. At follow-up visits, some of the participants voiced a preference for skipping some of these procedures which they had experienced adversely in the first visit (in these cases, the procedures in question were omitted). We feel it is important to share these experiences with the research community; while the multimodal approach provides a very rich and informative dataset, it also has limitations in terms of tolerability and ultimately may lead to higher-than-expected attrition. Third, this study was impacted by the COVID-19 pandemic. Recruitment and enrollment had reached their peak in early 2020, right before we needed to shut down the study temporarily. Although we were able to restart a few months later, like studies all over the world [42,43], this loss of momentum was extremely challenging for a longitudinal study that had already experienced challenges with recruitment and retention at its beginning. Finally, there was one accidental death in this sample which was related to a motor vehicle accident. Death in adolescents is a highly rare event, with suicide and motor vehicle accidents being the top causes of death in this age group [44].

### Limitations and Future Directions

While the current study provides valuable insights into the experiences of individuals who engage in NSSI and may inform future research and interventions in this area, several limitations inherent to this study should be considered. First, despite efforts to recruit a diverse sample of adolescents, the final sample was predominantly non-Hispanic or Latinx, White, and middle-class. Also, we only included individuals who were assigned female sex at birth. These factors limit the generalizability of the findings to other populations. Second, while the study was designed to collect data at three yearly time points, given the challenges discussed above with respect to attrition, there were variations in the time gaps between appointments. This will need to be carefully considered in designing all longitudinal analyses with this dataset. Third, the COVID-19 pandemic caused a significant interruption to the study, which could have influenced the results. For example, it has been repeatedly documented that adolescents experienced an increase in stress, anxiety, depression, and suicidal thinking after the onset of the pandemic [45,46]. This will also need to be considered when designing longitudinal analyses of these data. Fourth, as discussed above, the study encountered several issues retaining this high-risk sample. Due to dropout or missing data even when participants were retained (e.g., agreeing to participate in a visit but not completing all measures due to limited time or preference not to complete certain measures, or administrator error), there is significant missingness in the final dataset, especially at later time points. Fifth, the clinical findings here primarily rely on self-report measures. Participants may not accurately report their experiences and behaviors for a number of reasons, ranging from recall bias to the desire to appear a certain way to the research team, to the desire to avoid certain things being shared with parents or reported to authorities, to a desire to avoid further questioning and/or a longer visit. Self-reported data can also be influenced by individual differences in interpretation and understanding of the questions, which can affect the reliability and validity of the findings. In addition, self-reported data may not capture the full complexity of NSSI behaviors and related factors, such as the severity and frequency of self-harm. Sixth, while it appears that several participants shifted to the No NSSI group by the end of the study from the Mild, Moderate, and Severe NSSI groups, we did not collect systematic information on treatments other than medication management that could have facilitated this transition. Hence, we are unable to ascertain whether certain treatments like psychotherapy may have aided in reducing NSSI severity in these participants. Lastly, given the nature of recruitment and consent for participating in the study, most parents were aware that their adolescents had engaged in NSSI. However, previous studies have reported that most parents are unaware of their child’s NSSI [47,48]; as such, our study may have an overrepresentation of parental awareness of NSSI and represent a group of families who are willing to share their experiences of youth’s suffering.

## CONCLUSIONS

In conclusion, we present longitudinal clinical trajectories in adolescents with and without a history of NSSI. Notably, attrition rates were higher than expected. Adolescents with a history of NSSI appeared to have higher rates of psychopathology than those without, but we report an overall trend of decreasing severity of psychopathology over time. We hope these descriptive results will spark new ideas for questions that could be pursued with this rich dataset that includes multimodal brain, cognitive, and physiological stress response data.

## Supplementary Material

Supplementary file

## Figures and Tables

**Table 1.** Criteria for NSSI Group Assignment.

NSSI Group	Criteria

No NSSI	No history of NSSI
Mild NSSI	< 4 past episodes of NSSI with significant tissue damage OR an unlimited number of episodes with mild or no tissue damage^1^
Moderate NSSI	≥ 4 past episodes of NSSI with significant tissue damage AND < 1/month frequency
Severe NSSI	≥ 4 past episodes of NSSI with significant tissue damage AND ≥ 1/month frequency

*Note:* NSSI = Non-Suicidal Self-Injury.

1 We acknowledge that people who engage in self-injurious behavior with mild or no tissue damage are a tricky group to categorize. The definition of NSSI used in this study [25] requires tissue damage. Hence, we concluded that the aforementioned group were not technically engaging in NSSI but a different kind of, arguably milder, self-injurious behavior. Since we were trying to create a spectrum of severity of NSSI engagement, we decided to place these participants in the Mild NSSI group as they fell lower on the spectrum of severity than both the Moderate and Severe NSSI groups but higher than the No NSSI group.

**Table 2.** Sample Demographic Characteristics ^1^.

Variable	*N* = 164

Age in years ^2^, mean (SD)	14.97 (1.20)

No NSSI	14.88 (1.24)
Mild NSSI	14.90 (1.13)
Moderate NSSI	14.79 (1.24)
Severe NSSI	15.42 (1.08)

Ethnicity	

Hispanic or Latin(o/a)/Latinx	19 (11.6%)
Not Hispanic or Latin(o/a)/Latinx	145 (88.4%)

Race	

African American or Black	5 (3.0%)
American Indian/Alaska native	1 (0.6%)
Asian	4 (2.4%)
White	132 (80.5%)
Indigenous/Chicano	1 (0.6%)
More than one race	21 (12.8%)
African American or Black, White	8 (4.9%)
American Indian/Alaska Native, African American or Black, White	2 (1.2%)
American Indian/Alaska Native, Asian, White	1 (0.6%)
American Indian/Alaska Native, White	5 (3.0%)
Asian, White	3 (1.8%)
Native Hawaiian or other Pacific Islander, White	1 (0.6%)
Indigenous/Buryat, White	1 (0.6%)

Gender identity	

Female	118 (72.0%)
Male	5 (3.0%)
Nonbinary	8 (4.9%)
Other-Gender fluid	1 (0.6%)
Not reported	32 (19.5%)

Languages usually spoken at home	

English	152 (92.7%)
English and Spanish	5 (3.0%)
English and Other-American Sign Language	1 (0.6%)
English, Spanish, and Other-Portuguese	1 (0.6%)
Not reported	5 (3.0%)

Household income	

Under $5,000	1 (0.6%)
$10,000–$14,999	4 (2.4%)
$15,000–$24,999	4 (2.4%)
$25,000–$39,999	17 (10.4%)
$40,000–$59,999	11 (6.7%)
$60,000–$89,999	26 (15.9%)
$90,000–$179,999	63 (38.4%)
Over $180,000	32 (19.5%)
Unknown	1 (0.6%)
Not reported	5 (3.0%)

Parent or Guardian educational status	

First Parent or Guardian ^3^	
Junior high school (7th, 8th, 9th)	1 (0.6%)
Some high school (10th, 11th, 12th)	1 (0.6%)
High-school graduate (can be equivalency exam)	7 (4.3%)
Some college or technical school (at least one year)	26 (15.9%)
College graduate	66 (40.2%)
Graduate professional training (Master’s or above)	58 (35.4%)
Not reported	5 (3.0%)
Second Parent or Guardian ^4^	
Less than 7 years of school	2 (1.2%)
Junior high school (7th, 8th, 9th)	2 (1.2%)
Some high school (10th, 11th, 12th)	1 (0.6%)
High-school graduate (can be equivalency exam)	14 (8.5%)
Some college or technical school (at least one year)	33 (20.1%)
College graduate	58 (35.4%)
Graduate professional training (Master’s or above)	43 (26.2%)
Unknown	3 (1.8%)
Not reported	8 (4.9%)

*Note:* Data are *n* (%) of participants unless indicated otherwise. SD = Standard deviation.

1 T1 data were reported when available. T2 or T3 responses were used when T1 data were unavailable.

2 Age at T1 interview visit for the whole sample and by past-year NSSI group assigned at T1. Ages were similar across groups at T1: F(3, 159) = 1.896, *p* = 0.132.

3 First parent or guardian information was about the participant’s biological/adoptive mother (for 95.1% of participants), stepmother (0.6%), or foster mother (1.2%). The remaining 3.0% did not report any information on first parent or guardian.

4 Second parent or guardian information was about the participant’s biological/adoptive father (88.4%), stepfather (4.9%), grandfather (0.6%), other guardian - second mother (0.6%), or other guardian - adoptive mother (0.6%). The remaining 4.9% did not report any information on second parent or guardian.

**Table 3.** Rates of Psychiatric Diagnoses in the Sample According to the K-SADS-PL at Each Time Point.

Diagnosis	Time 1 (*N* = 164)	Time 2 (*N* = 124)	Time 3 (*N* = 94 ^1^)

Major Depressive Disorder	108	65	49
	90 current	37 current	31 current
	2 partial remission	20 partial remission	9 partial remission
	16 past	8 past	9 past

Persistent Depressive Disorder	14	9	8
	10 current	8 current	8 current
	2 past	1 past	
	2 status unsure		

Unspecified Depressive Disorder	1	0	0
	1 current		

Bipolar Disorder	0	3	2
		2 bipolar I, current, current depressive; 1 bipolar II, current, not specified	1 bipolar I, current, most recent manic; 1 bipolar II, current, current depressive

Generalized Anxiety Disorder	60	33	18
	41 current	18 current	13 current
	19 past	2 partial remission	1 partial remission
		13 past	4 past

Social Anxiety Disorder	25	25	14
	17 current	14 current	10 current
	8 past	2 partial remission	1 partial remission
		9 past	3 past

Separation Anxiety Disorder	21	4	1
	9 current	1 current	1 current
	12 past	3 past	

Panic Disorder	17	13	7
	8 current	3 current	3 current
	9 past	10 past	4 past

Post-traumatic Stress Disorder	36	31	24
	24 current	15 current	15 current
	3 partial remission	7 partial remission	3 partial remission
	9 past	9 past	6 past

Obsessive Compulsive Disorder	12	13	8
	12 current	10 current	7 current
		1 partial remission	1 partial remission
		2 status unsure	

Attention Deficit Hyperactivity Disorder	44	40	36
	39 current	34 current	31 current
	1 partial remission	5 partial remission	5 partial remission
	4 past	1 past	

Substance Use Disorder (other than tobacco use)	0	11	14
		9 current	13 current
		2 past	1 past

Eating Disorder	19	16	14
	18 current	14 current	9 current
	1 past	2 past	4 partial remission
			1 past

*Note:* K-SADS-PL = Kiddie Schedule for Affective Disorders and Schizophrenia for School-Age Children-Present and Lifetime Version.

1 Although 95 participants completed the clinical assessment at T3, the K-SADS-PL was not fully complete for one participant, so the diagnoses were not finalized before they chose to withdraw from the study.

**Table 4.** Rates of Any Psychiatric Diagnosis and Treatment by NSSI Group Across Time.

NSSI Group	Any Psychiatric Diagnosis, *N* (percentage)			Antidepressants, *N* (percentage)			Other Psychotropic Medications, *N* (percentage)			Seeing a therapist, *N* (percentage)		
	T1	T2	T3	T1	T2	T3	T1	T2	T3	T1	T2	T3
No NSSI	41 (46.1)	39 (62.9)	37 (61.7)	11 (15.1)	20 (32.3)	25 (41.7)	9 (12.3)	14 (22.6)	18 (30.0)	36 (49.3)	39 (62.9)	43 (71.7)
Mild NSSI	24 (88.9)	28 (87.5)	13 (100)	14 (51.9)	14 (43.8)	7 (53.8)	9 (33.3)	10 (31.3)	7 (53.8)	24 (88.9)	24 (75.0)	12 (92.3)
Moderate NSSI	32 (100)	13 (92.9)	10 (100)	16 (50.0)	11 (78.6)	9 (90.0)	9 (28.1)	10 (71.4)	5 (50.0)	31 (96.9)	12 (85.7)	9 (90.0)
Severe NSSI	30 (96.8)	16 (100)	10 (90.9)	18 (58.1)	11 (68.8)	5 (45.5)	13 (41.9)	9 (56.3)	6 (54.5)	31 (100)	15 (93.8)	10 (83.3)

*Note*: T1, T2, and T3 data were categorized based on past-year NSSI groups assigned at the respective time points. NSSI = Non-Suicidal Self-Injury.

**Table 5.** Severity of Depression Symptoms and Suicidal Thoughts and Behaviors Over Time.

Assessment	Time 1	Time 2	Time 3

CDRS-R, mean (SD), *N*	37.54 (17.29), *N* = 161	35.02 (14.65), *N* = 124	33.25 (14.58), *N* = 95
BDI-II, mean (SD), *N*	15.98 (14.29), *N* = 149	13.58 (11.01), *N* = 117	13.16 (10.82), *N* = 83
BSSI, mean (SD), *N*	4.82 (7.39), *N* = 158	2.59 (4.85), *N* = 116	2.43 (5.46), N = 81

*Note:* Ns differ from consort as some participants had incomplete or missing data. CDRS-R = Children’s Depression Rating Scale Revised, SD = Standard Deviation, BDI-II = Beck Depression Inventory-II, BSSI = Beck Scale for Suicidal Ideation.

**Table 6.** Invalid Responses on the PAI-A by Type of Invalidity and NSSI Group.

	No NSSI (Invalid/Total) ^1^	Mild NSSI (Invalid/Total)	Moderate NSSI (Invalid/Total)	Severe NSSI (Invalid/Total)

**Invalid Response** ^2^	**11**/137	**4**/47	**5**/32	**9**/36

Inconsistent	3	1	2	7
Infrequent	3	1	4	4
Negative Response Bias	0	2	0	1
Positive Response Bias	6	0	0	0

*Note:* NSSI = Non-Suicidal Self-Injury.

1 ‘Invalid’ refers to the number of invalid PAI-A responses in the respective NSSI group across all timepoints. ‘Total’ refers to the total number of PAI-A responses in the respective NSSI group across all timepoints.

2 Overall invalid responses number may not reflect total of four types above as some responses were invalid due to more than one indicator.

**Table 7.** PAI-A scores by NSSI Group Across Time.

Borderline Scores by NSSI Group					Clinical Impairment		
					Mild	Moderate	Severe

NSSI Group	Time Point	Mean	SD	*N*	60 ≤ T < 70	70 ≤ T < 81	T ≥ 81

No NSSI	1	46.7	10.2	52	5	1	0
No NSSI	2	46.5	8.5	39	4	0	0
No NSSI	3	46.7	7.7	35	2	0	0
Mild NSSI	1	60.7	10.7	18	7	2	1
Mild NSSI	2	52.1	8.5	16	3	0	0
Mild NSSI	3	53.1	9.6	9	3	0	0
Moderate NSSI	1	62.5	9.2	19	8	4	0
Moderate NSSI	2	55.5	13.9	6	0	0	1
Moderate NSSI	3	43	1.4	2	0	0	0
Severe NSSI	1	64.3	11.6	19	5	5	1
Severe NSSI	2	55.4	7.5	5	1	0	0
Severe NSSI	3	46.5	4.8	4	0	0	0

Depression Scores by NSSI Group					Clinical Impairment		
					Mild	Moderate	Severe

NSSI Group	Time Point	Mean	SD	*N*	60 ≤ T < 70	70 ≤ T < 84	T ≥ 84

No NSSI	1	50.8	12.7	52	7	4	1
No NSSI	2	43.9	8	39	1	1	0
No NSSI	3	43.9	4.8	35	1	0	0
Mild NSSI	1	66.7	10.5	18	6	7	1
Mild NSSI	2	47.9	6.4	16	0	0	0
Mild NSSI	3	49.7	10.3	9	3	0	0
Moderate NSSI	1	69.3	11.8	19	7	8	1
Moderate NSSI	2	50.2	9.2	6	0	0	0
Moderate NSSI	3	41.5	2.1	2	0	0	0
Severe NSSI	1	67.9	11.3	19	5	7	2
Severe NSSI	2	49.6	8.3	5	1	0	0
Severe NSSI	3	47.5	15	4	0	1	0

Anxiety Scores by NSSI Group					Clinical Impairment		
					Mild	Moderate	Severe

NSSI Group	Time Point	Mean	SD	*N*	60 ≤ T < 70	70 ≤ T < 81	T ≥ 81

No NSSI	1	52.5	11.7	52	8	5	0
No NSSI	2	45.5	8.2	39	3	0	0
No NSSI	3	46	7.6	35	2	0	0
Mild NSSI	1	61.7	12.7	18	5	3	2
Mild NSSI	2	49.5	7.2	16	2	0	0
Mild NSSI	3	51.2	7.7	9	2	0	0
Moderate NSSI	1	64.7	12.9	19	5	6	1
Moderate NSSI	2	51	8.4	6	1	0	0
Moderate NSSI	3	44	1.4	2	0	0	0
Severe NSSI	1	69.5	12.8	19	5	7	3
Severe NSSI	2	49.8	4.7	5	0	0	0
Severe NSSI	3	43	16.8	4	1	0	0

Suicide Scores by NSSI Group					Clinical Impairment		
					Mild	Moderate	Severe^1^

NSSI Group	Time Point	Mean	SD	*N*	60 ≤ T < 70	70 ≤ T < 86	T ≥ 86

No NSSI	1	50.3	12.8	52	2	5	1
No NSSI	2	44.2	8.5	39	0	1	0
No NSSI	3	44.8	5.8	35	1	0	0
Mild NSSI	1	64.5	13.5	18	5	7	0
Mild NSSI	2	47.6	6.8	16	0	0	0
Mild NSSI	3	49	8	9	1	0	0
Moderate NSSI	1	69.1	16.4	19	6	5	3
Moderate NSSI	2	49.5	8.2	6	0	0	0
Moderate NSSI	3	38.5	2.1	2	0	0	0
Severe NSSI	1	70.8	13.3	19	4	9	2
Severe NSSI	2	53.4	8.7	5	1	0	0
Severe NSSI	3	44.5	7.6	4	0	0	0

Mania Scores by NSSI Group					Clinical Impairment		
					Mild	Moderate	Severe

NSSI Group	Time Point	Mean	SD	*N*	55 ≤ T < 65	65 ≤ T < 73	T ≥ 73

No NSSI	1	43.3	7.9	52	4	0	0
No NSSI	2	44.9	9	39	7	0	0
No NSSI	3	46.7	9.7	35	5	2	0
Mild NSSI	1	43.3	9.1	18	1	1	0
Mild NSSI	2	52.8	11.5	16	6	0	2
Mild NSSI	3	52	8.5	9	3	0	0
Moderate NSSI	1	45.5	9.2	19	3	1	0
Moderate NSSI	2	54.8	8.9	6	4	0	0
Moderate NSSI	3	43	4.2	2	0	0	0
Severe NSSI	1	48.8	10.3	19	2	1	1
Severe NSSI	2	49.6	8.3	5	1	0	0
Severe NSSI	3	47.5	3.9	4	0	0	0

*Note:* PAI-A = Personality Assessment Inventory–Adolescent, NSSI = Non-Suicidal Self-Injury, SD = Standard Deviation.

1 One participant was not assigned a past-year NSSI group at T1 as they did not provide sufficient data for group assignment and was not included in this table: this participant scored above clinical threshold for suicidality at T1.

**Table 8.** CTQ Scores by Subscale Across Time.

CTQ Subscale	Time 1	Time 2	Time 3

Physical Abuse, Mean (SD), *N*	5.92 (2.14), *N* = 146	5.69 (1.63), *N* = 113	5.99 (2.24), *N* = 83
Emotional Abuse, Mean (SD), *N*	9.36 (4.60), *N* = 146	9.18 (4.55), *N* = 113	9.94 (5.26), *N* = 83
Sexual Abuse, Mean (SD), *N*	6.22 (3.68), *N* = 146	6.29 (3.29), *N* = 113	6.77 (3.83), *N* = 83
Emotional Neglect, Mean (SD), *N*	9.01 (3.82), *N* = 146	9.22 (4.40), *N* = 113	9.94 (4.35), *N* = 83
Physical Neglect, Mean (SD), *N*	6.66 (2.33), *N* = 147	6.37 (2.04), *N* = 114	6.77 (2.51), *N* = 83

*Note:* Ns differ from consort as some participants had incomplete or missing data. CTQ = Child Trauma Questionnaire, SD = Standard Deviation.

## Data Availability

The dataset described here is publicly available in the NIMH National Data Archive(NDA) under the number C2401.

## ABBREVIATIONS

ADHD: Attention Deficit Hyperactivity Disorder
BDI-II: Beck Depression Inventory-II
BSSI: Beck Scale for Suicidal Ideation
BRIDGES: BRain Imaging Development of Girls’ Emotion and Sel
CDRS-R: Children’s Depression Rating Scale, Revised
CTQ: Child Trauma Questionnaire
DBT: Dialectical Behavioral Therapy
GAD: Generalized Anxiety Disorder
K-SADS-PL: Kiddie Schedule for Affective Disorders and Schizophrenia for School-Age Children-Present and Lifetime Version
MRI: Magnetic Resonance Imaging
NDA: National Data Archive
NIMH: National Institutes of Mental Health
NSSI: Non-Suicidal Self-Injury
PAI-A: Personality Assessment Inventory–Adolescent
RDoC: Research Domain Criteria
SA: Suicide Attempt
SD: Standard Deviation
SI: Suicidal Ideation
SITBI: Self-Injurious Thoughts and Behaviors Interview
STB: Suicidal Thoughts and Behaviors
TSST: Trier Social Stress Test
