# Supplementary material for: Clinical Trajectories in Adolescents with and without a History of Non-Suicidal Self-Injury: The BRIDGES Longitudinal Study
Source: J Psychiatr Brain Sci. Author manuscript; Available in PMC 2023 Aug 15. (PMC10426765; doi:10.20900/jpbs.20230007)
Supplement: Supplementary file [file NIHMS1914336-supplement-Supplementary_file.pdf]

Supplementary Materials for “Clinical Trajectories in Adolescents With and Without a History of Non-Suicidal Self-Injury: The BRIDGES Longitudinal Study”

SECTION S1: RACE AND ETHNICITY DATA COLLECTION

The Office of Management and Budget standards for race and ethnicity categories were followed to collect the race and ethnicity information (Ethnicity categories: “Hispanic/ Latino(a)” and “Not Hispanic/Latino(a)”; Race categories: “African American or Black”, “American Indian/Alaska native”, “Asian”, “Native Hawaiian or other Pacific Islander”, and “White”). Responders were allowed to choose more than one of these categories. They were also provided the option to choose “More than one race”, “Other”, or “Don’t know”. Those selecting “More than one race” or “Other” were prompted to provide a write-in response to specify or explain their selection. Additionally, the parent and the participant were asked to write-in their ethnicity and race during the clinical diagnostic interviews at each time point.

Our group has decided to report monoracial, bi/multiracial status for each participant so the total in the tables for race results (Table 2) represents 100% of our participants rather than separately accounting for each multi-racial identity individually, which would result in >100% racial/ethnic status reported. This decision was made in an effort to more clearly delineate the unique identities of our participants [49].

Additionally, we acknowledge that some individuals and organizations have encouraged the use of “white,” given the ambiguity of white racial identity and the potential for unintentionally signaling white supremacist ideology. In understanding that discussions surrounding language use and racial/ethnic identity are fluid and evolving, we use “White” in this manuscript to be consistent with APA style [50].

SECTION S2: SUMMARY OF ADDITIONAL DATA COLLECTED AS PART OF THE BRIDGES STUDY

The BRIDGES (BRain Imaging Development of Girls’ Emotion and Self) study employed data from three assessment waves, each of which included three visits consisting of 1) consent, clinical assessment, and self-report measures, 2) modified Trier Social Stress Test (TSST), and 3) neuroimaging. Additionally, participants completed a cognitive battery either during the TSST or the neuroimaging visit. During the first visit of each wave, participants and their parents completed the Kiddie Schedule for Affective Disorders and Schizophrenia for School-Age Children-Present and Lifetime Version (K-SADS-PL) [18] and many self-report measures, including those described in the main text, in addition to some on emotion socialization, disordered eating, pubertal status, involvement in extracurricular activities, and rejection sensitivity.

The participants completed a modified version of the Trier Social Stress Test (TSST) during the second visit. They were asked to give an introduction speech and perform verbal arithmetic calculations while being observed by two examiners trained to remain neutral and to avoid giving reassurance or feedback (based on a validated measure of the procedure [51,52]) adapted from the classic TSST [53]. This procedure has been repeatedly used to reliably elicit stress responses in female youth [54]. Participants were scheduled to complete the TSST between 1pm and 4 pm and were instructed to not eat, drink, smoke, or brush their teeth within the hour prior to providing saliva samples.

Neuroimaging data was collected in the final visit. Brain scans were conducted using a Siemens 3 Tesla Prisma scanner and a 32-channel receive-only head coil, using the Human Connectome Project [55] multiband sequences. Structural scans were acquired using T1-weighted, multiecho Magnetization-Prepared RApid Gradient-Echo sequence and a T2-weighted Sampling Perfection with Application optimized Contrasts using different flip angle Evolution sequence. A series of functional MRI (fMRI) scans were obtained consisting of whole brain T2*-weighted functional volumes with 2 mm isotropic voxel

resolution using the HCP multiband echo planar imaging sequence. Participants first underwent a resting-state scan during which they were instructed to stay awake, focus their eyes on a fixation cross, and "not think about anything in particular." Participants also completed an emotion-face matching task in which they match either emotionally expressive faces (characterized by fear or anger) or neutral shapes [56]. The emotion-face matching task was conducted via E-prime software [57], and stimuli were projected onto a screen inside the bore of the scanner.

SECTION S3: ADDITIONAL SAMPLE DEMOGRAPHIC CHARACTERISTICS

Table S1. Additional Sample Demographic Characteristics[1]

Variable	N (%) (N=164)
Adopted	
Yes	9 (5.5%)
No	150 (91.5%)
Not reported	5 (3.0%)
Current living situation	
With parents (at least one parent)	158 (96.3%)
With relatives other than parents - grandmother	1 (0.6%)
Not reported	5 (3.0%)
Country of birth	
Australia	1 (0.6%)
Brazil	1 (0.6%)
Canada	1 (0.6%)
China	1 (0.6%)
Guatemala	1 (0.6%)
India	1 (0.6%)
Nicaragua	1 (0.6%)
Russia	2 (1.2%)
United States of America	150 (91.5%)
Not reported	5 (3.0%)
Type of school	
Regular public school (includes schools for the "gifted," charter and magnet schools, etc.)	139 (84.8%)
Regular private school (including Catholic, Jewish, or other parochial schools, etc.)	12 (7.3%)
Special school for children with emotional/behavioral problems, learning problems, etc. (includes full-time program located within a larger school)	8 (4.9%)
Not reported	5 (3.0%)
Parent or Guardian occupational status	
First Parent or Guardian[2]	

Full-time, paid employment	82 (50.0%)
Part-time, paid employment	30 (18.3%)
Homemaker	9 (5.5%)
Full-time student	3 (1.8%)
On disability	3 (1.8%)
Unemployed	5 (3.0%)
Retired	4 (2.4%)
Other	4 (2.4%)
More than one occupational status	18 (11.0%)
Not applicable	1 (0.6%)
Not reported	5 (3.0%)
Second Parent or Guardian[3]	
Full-time, paid employment	122 (74.4%)
Part-time, paid employment	5 (3.0%)
Homemaker	2 (1.2%)
On disability	2 (1.2%)
Unemployed	7 (4.3%)
Retired	2 (1.2%)
Other	8 (4.9%)
More than one occupational status	5 (3.0%)
Unknown	3 (1.8%)
Not reported	8 (4.9%)
Parent or Guardian marital status	
First Parent or Guardian	
Married to child's parent	97 (59.1%)
Married to someone other than child's parent	16 (9.8%)
Separated	5 (3.0%)
Divorced	26 (15.9%)
Widowed	3 (1.8%)
Never married	9 (5.5%)
Other	3 (1.8%)
Not reported	5 (3.0%)
Second Parent or Guardian	
Married to child's parent	103 (62.8%)
Married to someone other than child's parent	13 (7.9%)
Separated	1 (0.6%)
Divorced	22 (13.4%)
Widowed	1 (0.6%)

Never married	11 (6.7%)
Other	4 (2.4%)
Not reported	9 (5.5%)

Note. Data are n (%) of participants unless indicated otherwise. SD = Standard deviation.

[1] T1 data were reported when available. T2 or T3 responses were used when T1 data were unavailable.

[2] First parent or guardian information was about the participant's biological/adoptive mother (for 95.1% of participants), stepmother (0.6%), or foster mother (1.2%). The remaining 3.0% did not report any information on first parent or guardian.

[3] Second parent or guardian information was about the participant's biological/adoptive father (88.4%), stepfather (4.9%), grandfather (0.6%), other guardian - second mother (0.6%), or other guardian - adoptive mother (0.6%). The remaining 4.9% did not report any information on second parent or guardian.

SECTION S4: CHILDHOOD TRAUMA SCORES FOR RETURNING PARTICIPANTS AND DROPOUTS

Figure S1. Emotional, Physical, and Sexual Abuse Scores on the CTQ for Returning Participants and Dropouts

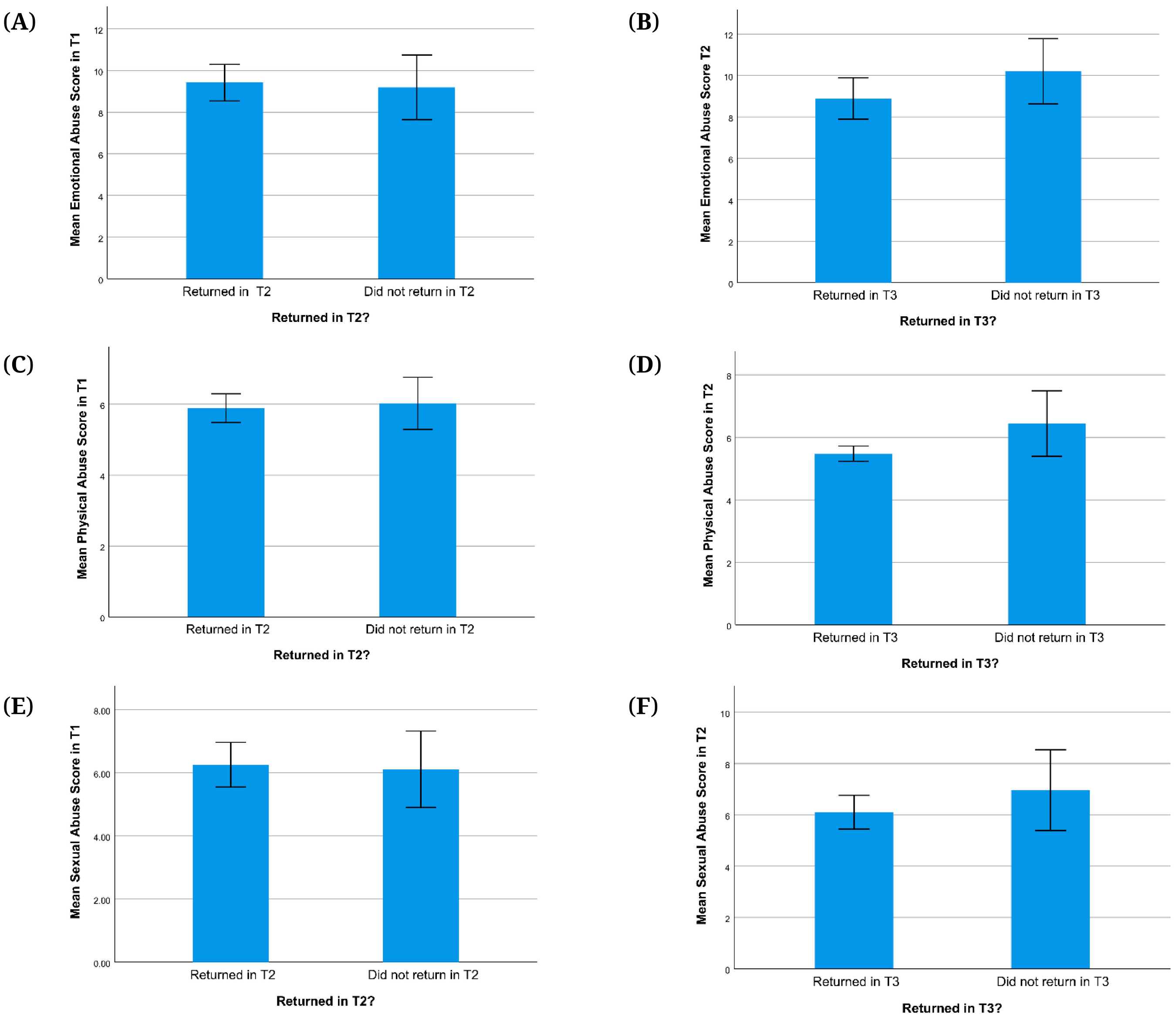

Note. Error bars are +/- 2 standard error. CTQ=Child Trauma Questionnaire. (**A**) Mean T1 CTQ emotional abuse scores for participants who returned in T2 and those who did not. (**B**) Mean T2 CTQ emotional abuse scores for participants who returned in T3 and those who did not. (**C**) Mean T1 CTQ physical abuse scores for participants who returned in T2 and those who did not. (**D**) Mean T2 CTQ physical abuse scores for participants who returned in T3 and those who did not. (**E**) Mean T1 CTQ sexual abuse scores for participants who returned in T2 and those who did not. (**F**) Mean T2 CTQ sexual abuse scores for participants who returned in T3 and those who did not.

Figure S2. Emotional and Physical Neglect Scores on the CTQ for Returning Participants and Dropouts

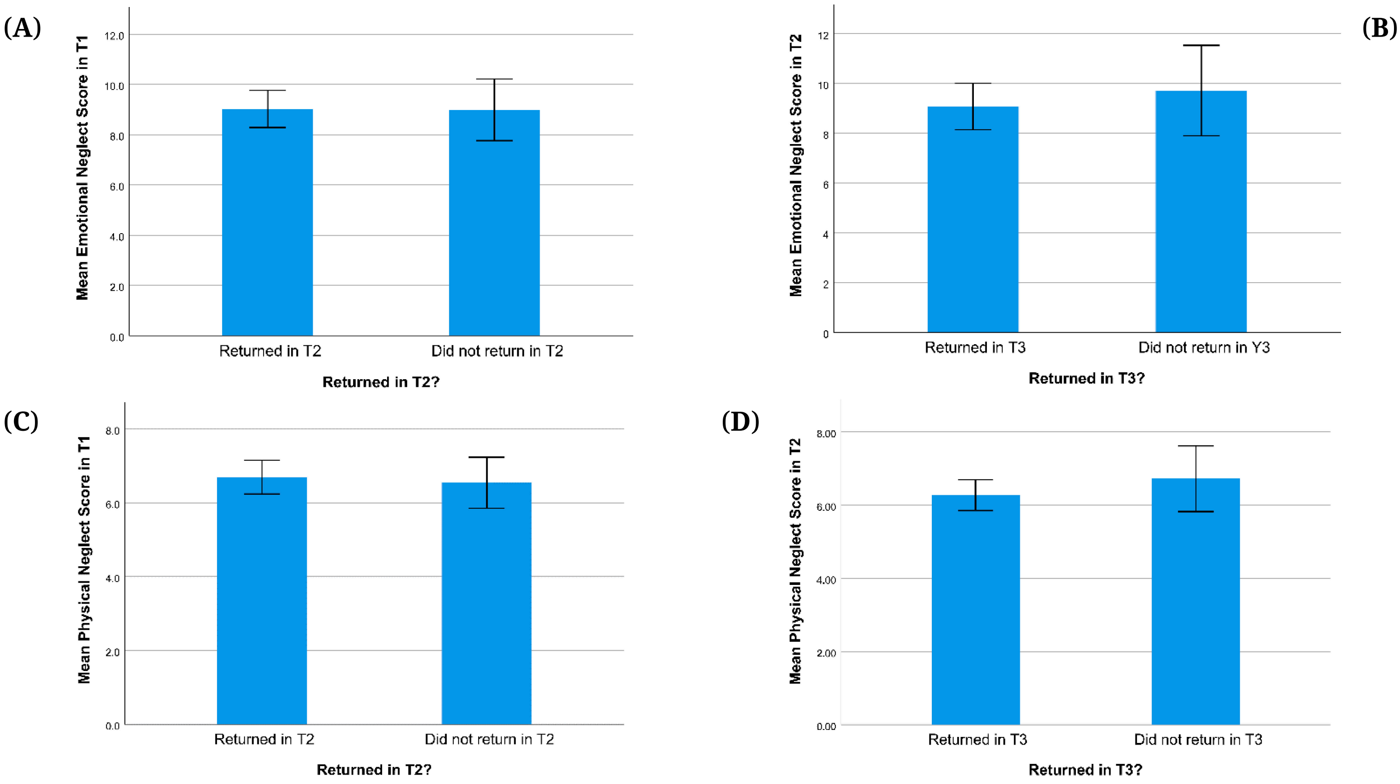

Note. Error bars are +/- 2 standard error. CTQ=Child Trauma Questionnaire. (**A**) Mean T1 CTQ emotional neglect scores for participants who returned in T2 and those who did not. (**B**) Mean T2 CTQ emotional neglect scores for participants who returned in T3 and those who did not. (**C**) Mean T1 CTQ physical neglect scores for participants who returned in T2 and those who did not. (**D**) Mean T2 CTQ physical neglect scores for participants who returned in T3 and those who did not.

SECTION S5: PATTERNS OF CHANGES IN DEPRESSION SEVERITY (BDI-II) AND SUICIDAL IDEATION (BSSI) SCORES

As with CDRS-R scores, linear multi-level regressions assessed how study visit time point, participant age, and duration of study participation were associated with trajectories of BDI-II and BSSI scores. Models were fit using R packages lme4 [58] and lmerTest [59].

Depression Severity: BDI-II

Similar to CDRS-R scores, BDI-II scores varied between- and within- participants at intake and across study participation (Figure S3A). However, unlike CDRS-R scores, BDI-II scores also had age-varied

developmental trends, with younger participants at intake showing decreases in overall BDI-II scores, and older participants showing little change or slight increases (Figure S3B). As with CDRS-R scores, BDI-II scores showed varied longitudinal trends based on age at study intake. On average, participants who were younger at intake saw decreases in their total BDI-II scores across study duration, whereas participants who were older at intake saw no change or small increases in their BDI scores over the course of study participation (Figure S3B).

Figure S3. Change Over Time in BDI-II Scores and Effect of Age at Start
(A)

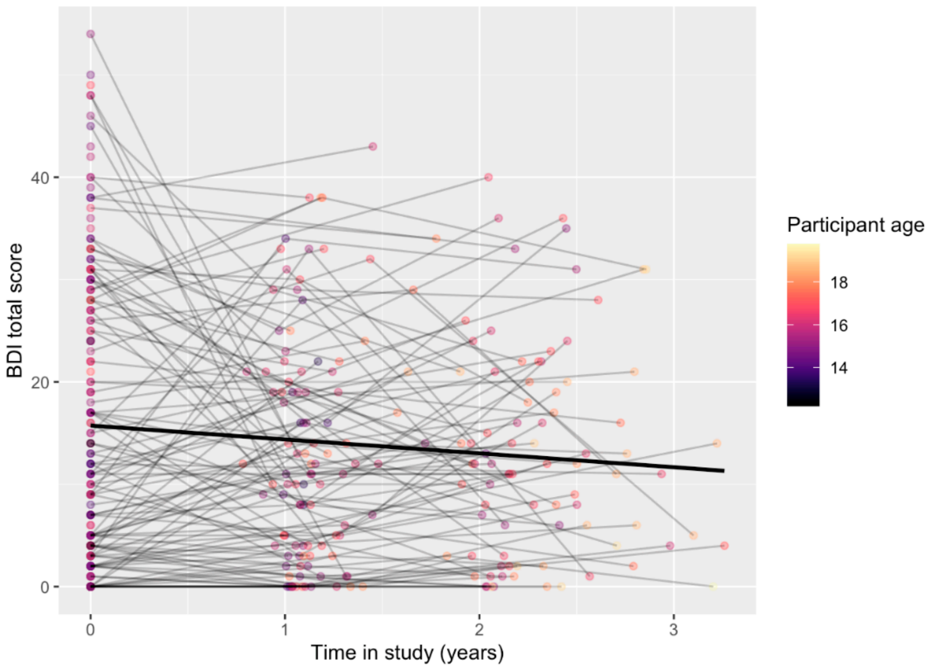

(B)

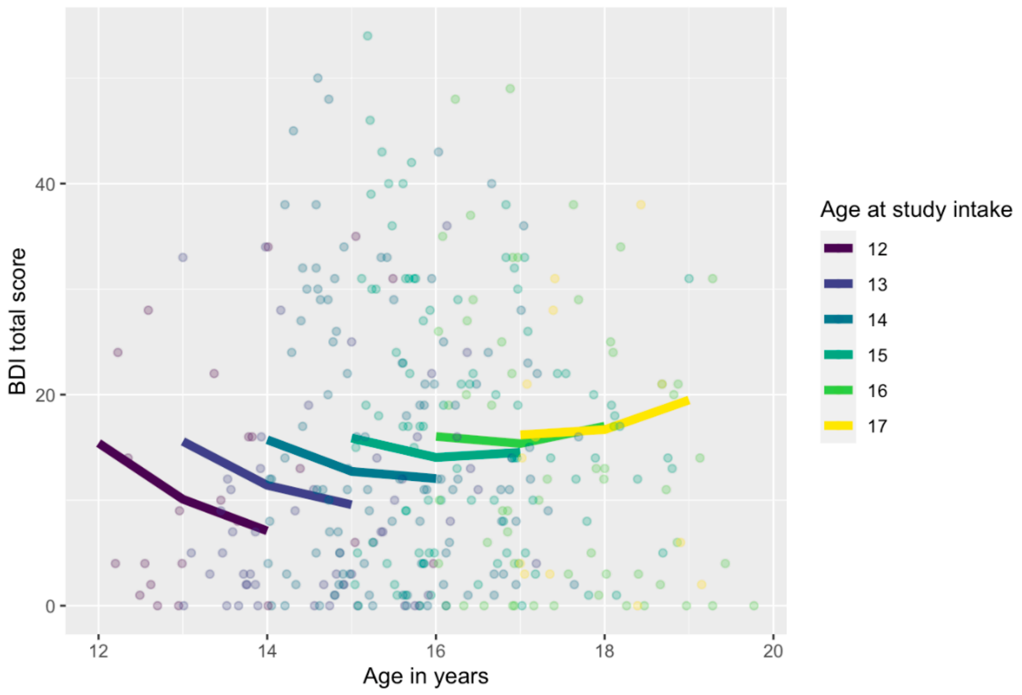

Note. BDI-II=Beck Depression Inventory-II. (**A**) Spaghetti plot showing change over time in BDI-II scores (**B**) Effect of age-at-start on BDI-II Scores: Data points represent raw participation data with predicted fitted trajectories by age group at intake overlaid.

Suicidal Ideation: BSSI

As seen in the figure below (Figure S4), BSSI scores at all three time points shows significant positive skew (i.e., many scores of 0). After accounting for inter-individual variation in baseline BSSI scores, change in BSSI scores was best modeled with a quadratic effect of time in the study (as seen in Figure S5). In the full sample, this equated to an average mean BSSI score of 4.49 at study intake, with scores decreasing until ~1.75 years into study participation, then increasing again (Figure S5A). Similarly, when only non-zero scores are considered, the average mean BSSI score at intake was 10.11, with scores decreasing until ~1.5 years into study participation, then increasing again (Figure S5B).

Figure S4. Distributions of BSSI Scores at Each Study Visit Time Point

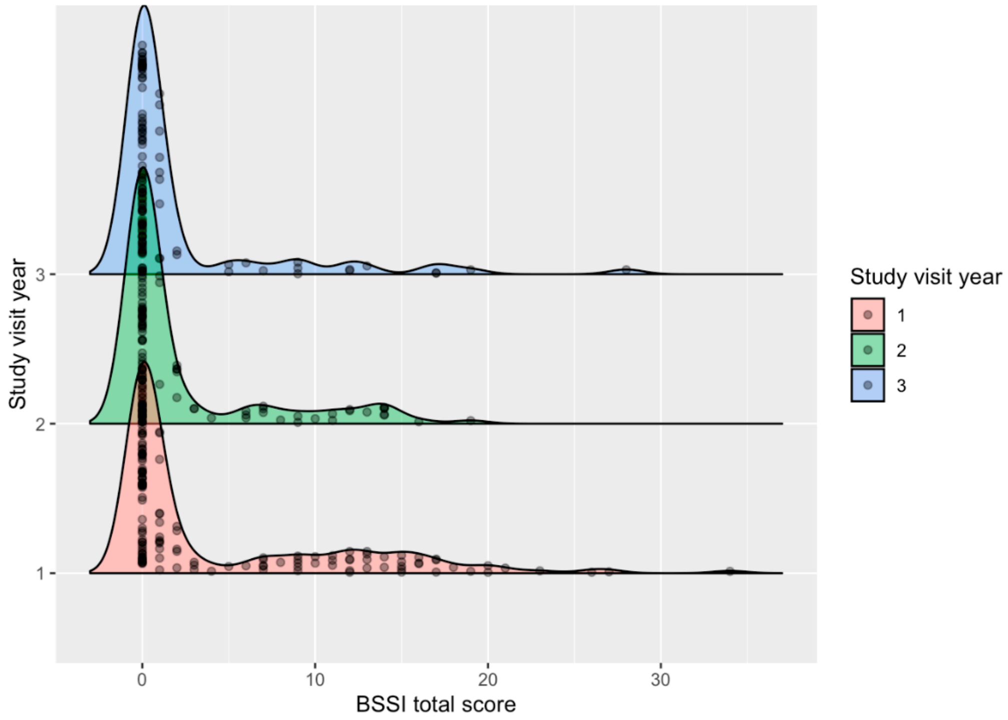

Note. BSSI=Beck Scale for Suicidal Ideation.

Figure S5. Predicted trajectories of BSSI scores with raw participant data.

(A)

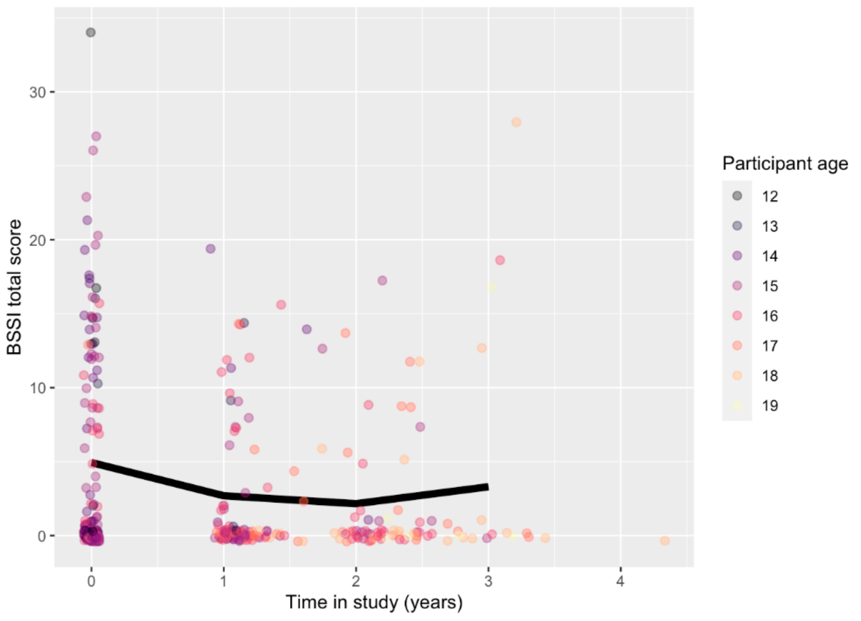

(B)

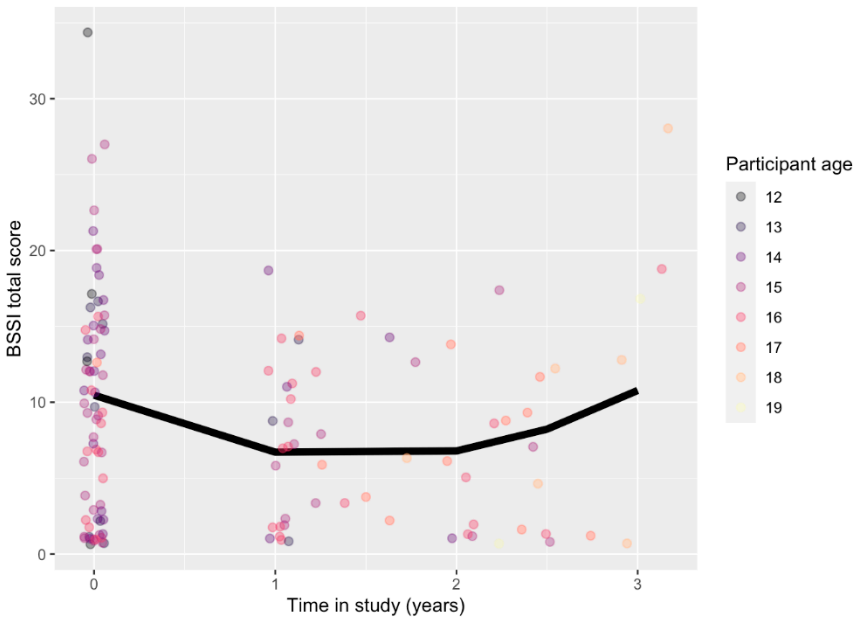

Note. BSSI=Beck Scale for Suicidal Ideation. (**A**) Predicted trajectory for all visits (**B**) Predicted trajectory as predicted using only non-zero BSSI scores.

SECTION S6: QUALITATIVE REASONS PROVIDED BY PARTICIPANTS IN THEIR OWN WORDS FOR SUICIDAL IDEATION, SUICIDE PLAN, SUICIDE ATTEMPT, NSSI THOUGHT, AND NSSI ENGAGEMENT AT TIME 1

Why do you think you have thoughts of killing yourself?

Don't Know

- I don't know
- not sure
- Not sure
- Unknown
- She doesn't know
- No clue
- IDK
- Don't know
- IDK
- Unknown
- Idk
- not sure, impulsive thought
- Don't know - no guess
- Honestly I don't know
- I don't know, just be easier
- Don't know
- Not sure
- don't know, I like my life and friends
- idk, it just started i guess. idk. i just kind of came in phases, and stuff that would happen that would cause it, or without warning.
- not sure, stress from school?; idk
- No idea; I don't know.

Feeling sad / depressed / bad feelings

- I want to stop feeling sad
- depression and anxiety
- Big emotions that I'm having
- unhappy with how things going
- It would be easier to not have to deal with stressful things.
- b/c really sad
- not sure - overwhelming feeling, not wanting go on, feeling stuck and useless
- Depression
- B/c I was tired of feeling that way
- A lot of depression, anxiety, mental illness
- Tired of everything
- "I was really depressed"
- Feel done everything, tired emotionally
- I was depressed
- Feeling really depressed
- B/c depressed
- overwhelmed feels like failure - don't know how to fix, unsafe life unsafe
- Think maybe bcos of depression
- B/c sad
- Depression
- really sad + wouldn't be worse than this

- Because I was depressed
- don't feel good
- She didn't know how to deal with stress and got really overwhelmed.
- Really upset about things/situations
- Going through a rough time + going through physical/emotional changes, growing up - took it badly*
- Felt bad outweighed good, felt low, no happiness, couldn't handle it
- Knew that I wanted to, was depressed/ sad
- I was angry at myself and thought it would be easier.*
- I am depressed and don't know how to handle it
- Because I don't feel happy or good enough*
- Don't know- sadness and only option from TV shows suicide references*
- not sure, impulsive thought or b/c depression*
- Life stressors; Sometimes they just happen
- Very sad, At the time, she was very attached to girlfriend and had hard time when girlfriend was upset.*
- B/c I'm sad
- Honestly I don't know, probably because social situations really stress me out.*
- Cause I was sad
- Because I have depression, poor self worth*
- Because feel sad and anxious
- So stressed out, didn't know how to cope
- A really rough day, or something dramatic happened.
- Right now, mostly school. General feelings of unhappiness. "don't really care anymore"
- because i'm depressed, because i feel bad all the time, because my dad hates me, because I'm swamped with schoolwork... a lot of reasons; i feel like a disappointment*

Low self-esteem / Self-hatred / unhappiness with body

- I want to stop feeling sad b/c don't like to live in the body I have*
- Because think of myself as disgusting
- not sure - overwhelming feeling, not wanting go on, feeling stuck and useless
- b/c felt hopeless & unsuccessful*
- overwhelmed feels like failure - don't know how to fix, unsafe life unsafe*
- because I have...part of it is my low self esteem, hard time liking self
- bc something triggered a bunch self-hatred
- Because I was depressed, didn't like myself, didn't appreciate life*
- How society thinks women should be- a certain description of how they want people to look- no fat*
- Because of the way my brain is wired. Because of my traumas- I don't think anyone wold like their body after someone defiled it.*
- Going through a rough time + going through physical/emotional changes, growing up - took it badly*
- felt worthless, like the world didn't need her
- I was angry at myself and thought it would be easier.*
- Unworthiness, feels unloved/cared about, would anybody notice if she was gone
- Because I don't feel happy or good enough*
- Felt like she wasn't good enough for herself, life had no meaning.*

- Disappointment in myself and everything doesn't feel like it's worth it.*
- Felt like not good enough for anyone; All would be better off if not there.*
- Because I have depression, poor self worth*
- Felt awful about myself
- Didn't view self in a + way, didn't think missed/needed
- I didn't like the way i looked. Early bloomer
- she feels like a failure, the world is bad and she cant do anything but it*
- There's something wrong with me. I don't want to be alive
- felt wasn't good enough, couldn't do anything right, no reason to live*
- feel like I deserve it
- feel hopeless. Doesn't want to become an adult; Thought she wasn't good enough and that nobody accepted her.*
- Because she doesn't feel worthy and doesn't like herself.
- because i'm useless
- idk... i seem to disappoint everyone around me and myself. i don't see how my presence would benefit or bring anyone anything positive
- i don't know, i struggle with self-esteem and i'm very hard on myself, just don't feel like I belong or that i'm good enough
- because i'm depressed, because i feel bad all the time, because my dad hates me, because I'm swamped with schoolwork... a lot of reasons; i feel like a disappointment*
- I don't know. I'm not really going anywhere. My body doesn't really work. It's better than a lot of people's, but it still kind of sucks
- Feeling really isolated and like I didn't matter, no one would care if I wasn't there

Brain

- "Because of the way my brain is wired. Because of my traumas- I don't think anyone wold like their body after someone defiled it.*
- Because think better off dead, and chemicals in her brain that cause suffering.*

Hopelessness / Life has no meaning / no future

- didn't think life had a point
- Everything is kind of pointless anyway
- b/c felt hopeless & unsuccessful*
- Afraid of future, won't be able to do things; really sad + wouldn't be worse than this*
- Because I was depressed, didn't like myself, didn't appreciate life*
- No hope
- at the time things were at the worst with parents, did not see a future or place for myself*
- Felt like she wasn't good enough for herself, life had no meaning.*
- Because think better off dead, and chemicals in her brain that cause suffering.*
- Disappointment in myself and everything doesn't feel like it's worth it.*
- I'm not satisfied with my life, don't know where it's going, uncertainty
- she feels like a failure, the world is bad and she cant do anything but it*
- felt wasn't good enough, couldn't do anything right, no reason to live*
- "don't really care anymore"
- feel hopeless. Doesn't want to become an adult; Thought she wasn't good enough and that nobody accepted her.*

- Sometimes it feels like I don't have anyone in my corner so there is no point to living. *

Relationships
- Dad, voices - command, 2-3 attempts b/c of voices*
- lots of conflict with family, thought it'd be better for them without her and bullying*
- at the time things were at the worst with parents, did not see a future or place for myself*
- Very sad, At the time, she was very attached to girlfriend and had hard time when girlfriend was upset.*
- because i'm depressed, because i feel bad all the time, because my dad hates me, because I'm swamped with schoolwork... a lot of reasons; i feel like a disappointment*
- Sometimes it feels like I don't have anyone in my corner so there is no point to living.

Psychosis
- Dad, voices - command, 2-3 attempts b/c of voices

An Escape / "only option"
- an escape
- A few different reasons, I feel like it was my only option it would have been an easy way to escape everything
- Don't know- sadness and only option from TV shows suicide references*
- A lot of pain and things to escape from
- Felt like couldn't do everything being asked of her
- Didn't like situation, wanted to get out
- Life is really difficult. idk, ... feel trapped, feels like only escape.

Loss
- bullying and deaths in life of loved ones*
- Because Dad died- exposure- seemed normal

Marginalized/ discrimination / rejection
- uncomfortable with self-identity/gender, bullying
- When people avoid me in hallway etc.
- because s/t friends said I was too slow or not good at a specific subject
- bullying and deaths in life of loved ones*
- How society thinks women should be- a certain description of how they want people to look- no fat*
- lots of conflict with family, thought it'd be better for them without her and bullying*

Feeling like a burden
- lots of conflict with family, thought it'd be better for them without her and bullying*
- Felt like not good enough for anyone; All would be better off if not there.*
- Used to think about them a lot more so now her brain just goes to them. If someone gets mad at her or if something at school happens. "sometimes i just feel like it would be a lot easier for other ppl if i wasn't here to mess things up. but i just think that when i'm really upset. but right now i know it would do more damage."

Why do you think you make suicide plans?

Don't Know

- I don't know
- IDK
- I don't know, but I know why I never went through with it - no guess
- "IDK - they make you feel better"
- Don't know
- i don't even know, mostly impulsive at the time.
- to go through with them, I'm not sure*

Feeling sad / depressed / bad feelings/ stress

- Felt guilty
- easier to not deal with stress
- really sad, don't want to be alive
- Because life was too stressful, didn't want to deal with it anymore
- She was really upset
- She wanted "out" of the stress in her life.*
- To get out of stressful life situations*
- More of an overreaction to going through a rough time
- I just don't know how to deal with my depression
- Because I'm depressed
- Same as suicidal thoughts- dealing w/sadness and an escape*
- To stop feeling sad/low.
- Assume it's because she was sad b/c that's the easy answer.
- It's hard to remember specifically, I think it was to stop things in my head from thinking bad things*
- emotional pain and distress has consumed all of me and taken away all my happiness and joy w/ a final triggering moment. like a shitty day or bad convo w/ parents, her being upset w/ something w/ relationships.*
- feeling very sad, hopeless, worthless; grades were plummeting; dad is the worst; no one likes me; what's the use of being here?*

Low self-esteem / Self-hatred / unhappiness with body

- I don't want to have to deal with my own emotions or have others see me dealing with them.
- unhappy, deserve it, self hate
- hopeless, afraid, self conscious, scared, tired of struggling.*
- I thought I wasn't important, no purpose to suffer thru what I'm going thru*
- feeling very sad, hopeless, worthless; grades were plummeting; dad is the worst; no one likes me; what's the use of being here?*
- others better off without me, confused about identity*

Brain

- distract the mind
- It's hard to remember specifically, I think it was to stop things in my head from thinking bad things*

Hopelessness / Life has no meaning / no future/ want to die

- I didn't want to exist anymore
- B/c wanted to die

- hopeless, afraid, self conscious, scared, tired of struggling.*
- living way I live and my mental state is so unbearable, I don't know what other option I have*
- Tired of everything
- done - over it. Didn't think there was a point to living at that time.
- I don't want to be alive
- Because wanted to die so bad.*
- because i think i'm going to be better off dead/ like that*
- because i wanted to die

Relationships

- peer relationships
- To send a message or escape and not have to deal with people or thoughts- or because of parents and grandpa*
- emotional pain and distress has consumed all of me and taken away all my happiness and joy w/ a final triggering moment. like a shitty day or bad convo w/ parents, her being upset w/ something w/ relationships.*
- feeling very sad, hopeless, worthless; grades were plummeting; dad is the worst; no one likes me; what's the use of being here?*

Psychosis

- easier, voices, escape*

An Escape / "only option"

- easier, voices, escape*
- way to escape ("get away from everything")
- living way I live and my mental state is so unbearable, I don't know what other option I have*
- Cause I wanna get away from the pain
- She wanted "out" of the stress in her life.*
- b/c of what she was going through- felt like someone wanted bad things to happen - fix pain - fix others lives- don't have to worry about her "Because I didn't want to live anymore"*
- To get out of stressful life situations*
- To get away, could start over and start something new, end bad problems
- I didn't want to deal with anything that was happening in my life anymore
- Same as suicidal thoughts- dealing w/sadness and an escape*
- As a way to cope, way to get out of it
- To send a message or escape and not have to deal with people or thoughts- or because of parents and grandpa*
- As an escape

Feeling like a burden

- easier to not deal with stress
- b/c didn't want to deal with anyone
- I don't want to have to deal with my own emotions or have others see me dealing with them.*
- b/c of what she was going through- felt like someone wanted bad things to happen - fix pain - fix others lives- don't have to worry about her "Because I didn't want to live anymore"*
- I thought I wasn't important, no purpose to suffer thru what I'm gong thru*
- because i think i'm going to be better off dead/ like that*

- because I felt like that was where I was at, life was just going to get worse and I couldn't handle it anymore, I'd be better off not to bother people, just a problem for other people
- others better off without me, confused about identity*

Just in case/ intent on acting upon them/ control

- Just in case I actually want to
- to make sure everythings going to work or how it would happen
- Bc I had intent on acting on them
- to go through with them, I'm not sure*
- 'Think about it because I know it's there and I have the option'
- Because easier to plan so she knows if it's possible.
- Because when I feel stuck and don't know what to do, you could just not do it and die.
- Makes me feel better to know that I can do it
- Serious about killing herself
- so that i know what to do if i do attempt , i will end up dead because that's the intention.
- Because wanted to die so bad.*
- makes me feel a little bit in control, feel really out of control and making a plan even if i'm not going to follow it makes me feel in control
- feels stable

Why do you think you make suicide attempts?

Don't Know

- IDK
- No idea
- IDK
- Don't know

Feeling sad / depressed / bad feelings

- feel bad, hate
- suffer
- A lot of pain and struggling to keep myself healthy
- Really stressful, filled with many emotions
- Being off meds, depression
- to get out of bad things happening in life
- Nobody knew I was depressed so I felt super alone, was sick of hiding things, didn't have help
- Because I wanted to put an end to how I was feeling, so people would realize that words hurt
- Sadness and escape
- I was angry and didn't feel like anyone liked me
- Because I'm too scared... want to die but doubt behind it
- didn't want to have to be a burden on other people's lives. also was feeling so terrible at that time.
- Hurting
- to get rid of negative feelings.

Low self-esteem / Self-hatred / unhappiness with body

- Because I don't feel worth it
- A lot of pain and struggling to keep myself healthy

- Didn't feel worthy
- I was angry and didn't feel like anyone liked me
- her dad lying, feels she deserves it

Hopelessness / Life has no meaning / no future/ want to die

- didn't want to live anymore
- guilt and hopelessness
- Don't want to be alive
- b/c don't want to live
- same as before, life being unbearable, not able to live as self
- Tired of everything
- I'm tired and don't want to be around anymore
- Feeling hopeless
- Didn't feel worthy
- Everything was really bad, had nothing good
- Had nothing good

Relationships

- peer exclusion, relationship
- Just got out of relationship, took it really hard, things built up
- Because I wanted to put an end to how I was feeling, so people would realize that words hurt
- felt like everyone would be better and dad told patient to
- I was angry and didn't feel like anyone liked me
- her dad lying, feels she deserves it
- mostly school stuff, everything going on. so much stuff going on.

Psychosis

- control, voices, overwhelming emotions, numb/need to feel

An Escape / "only option"/ Get away

- escape responsibilities, how to fix things
- Tired of being "depressed" and angry
- really done - didn't want to do anything anymore
- I'm tired and don't want to be around anymore
- Because I wanted to put an end to how I was feeling, so people would realize that words hurt
- Sadness and escape
- To get out of this place, to feel something.
- Felt like only option, felt like nothing was helping or working
- Because when I feel stuck/trapped I don't know what to do.
- To escape
- this specific attempt: so she didn't have to live in the future and the unknown.
- To try to escape pain.
- to get rid of negative feelings.
- the idea of not existing seems better than being here
- just felt so trapped.
- just really felt like no one cared if she was there or not, it was just the worst she had ever felt, just needed to get away

Marginalized/ discrimination / rejection

- I was angry and didn't feel like anyone liked me

To feel something/ Numb

- To get out of this place, to feel something.
- Really numb at the time, didn't have any emotions or feelings.

Feeling like a burden

- Because she "doesn't" want to live anymore. Felt guilty and selfish.
- felt like everyone would be better and dad told patient to
- the idea of not existing seems better than being here
- just really felt like no one cared if she was there or not, it was just the worst she had ever felt, just needed to get away
- didn't want to have to be a burden on other people's lives. also was feeling so terrible at that time.

Just in case/ intent on acting upon them/ control

- bc I had intentions to die
- To die

Lack of Support or Help

- Nobody knew I was depressed so I felt super alone, was sick of hiding things, didn't have help
- Felt like only option, felt like nothing was helping or working
- Because when I feel stuck/trapped I don't know what to do.
- just felt so trapped.

Why do you think you have thoughts of engaging in NSSI?

Don't Know

- Not really sure
- IDK
- Have no clue - don't even know why did it
- Uknown
- No idea
- IDK
- I don't know
- Doesn't know
- Doesn't know
- I don't know
- Don't know
- Idk, idk

Feeling sad / depressed / bad feelings – coping / relieve stress / pain

- Try to make self feel better.
- feeling bad, self hate
- cope w/ things- problems w/ bad feelings
- B/c dep[ression]
- feel ST, dad, voices, emotions

- Depression; self-loathing
- Sad, stressed
- to relieve some of the tension of life and its hardships
- "Because I don't necessarily want to die; I want to live but I just want to stop hurting."
- Because high anxiety, overwhelmed
- Stress, depressed
- Stressed
- Really stressed
- To help cope with the emotional/ distress that I feel
- Because momentarily it helps reduce emotional pain
- Super stressed and unhappy that couldn't help someone.
- It depends, really distressed, bored, interpersonal/school stuff, stress, feeling trapped.
- I was either bored or sad or mad
- feel guilty, really low, angry
- Situational stress
- i can't keep up w/ what's going on, no other way for me to express what's going on inside. way of expressing the intensity of what's going on.
- I am not sure, when something hard, anything goes wrong, how started to cope
- cope w/ things- problems w/ bad feelings
- Instinct of mine, like a habit, that's how I deal with stress sadly.
- Because it was a way of contraband letting out emotions, felt like I had to, it feels good when you need that
- to relieve some of the tension of life and its hardships
- Started out as a stress relief - now has "cravings" and "urges"
- As a way to release pain or emotion
- A way to cope with what she was going through
- To help cope with the emotional/ distress that I feel
- Because momentarily it helps reduce emotional pain
- Makes me feel something, forget or cope
- "It relieves stress"
- Release stress. stress, just don't know what to do.
- impulsive thing, in a bad mental spot
- I am not sure, when something hard, anything goes wrong, how started to cope
- It depends, really distressed, bored, interpersonal/school stuff, stress, feeling trapped.
- Doesn't know- her friends were all going through a lot but she isn't sure
- mostly school stress and stuff w/ friends
- if I knew, I'd be better. probably because I just don't like myself at all. People being rude and saying stuff that's not very nice. Having problems with family and friends.

Low self-esteem / Self-hatred / unhappiness with body

- b/c I hate myself so much, I want to hurt myself, I don't know if that make sense
- wanting to feel something and feeling like deserve it
- feeling bad, self hate
- thought I deserved it
- She wants to hurt herself but she doesn't want to die.
- Depression; self-loathing

- Because of body image and self hatred
- Because it was a way of contraband letting out emotions, felt like I had to, it feels good when you need that
- Usually because of anger at myself, like I need to be punished
- self-worth, "I deserve this because I'm not good enough" - punishment. And a little trying to feel something
- felt like she deserved it or needed to feel something
- Feels like she deserves the pain, like she isn't good enough and deserves punishment
- Retribution, punishment- guilt
- feel guilty, really low, angry
- idk i guess as a way to evoke punishment for mistakes, although that is a terrible way to deal with mistakes
- feel like I deserve it
- it makes me feel less guilty, actually feel something. relieve emotional pain as physical pain
- Don't know but maybe because I like the pain and because she is pretty and thinks that is all people see and it hurts
- if I knew, I'd be better. probably because I just don't like myself at all. People being rude and saying stuff that's not very nice. Having problems with family and friends.
- i think i should suffer because i'm the worst person in the world
- I convince myself I am a bad person.

Brain

- I want to feel physical pain because I feel pain in my brain I (think?).
- when i'm really upset, i just.. my brain feels like i'm going crazy ,and it's the only thing i've done that can help me not.. that can soften the blow a little bit. i know it has consequences but IDK what would happen if i didn't do it sometimes. i go wild in my brain.
- Release endorphins

Hopelessness / Life has no meaning / no future

- Cause I had no purpose

Relationships

- "Thinking what will happen." "How my parents would feel and stuff
- Bullying
- feel ST, dad, voices, emotions
- Idk....things happen, people do things to me, attention for them to stop
- Family; grandma died; abusive father; "just really down"
- Caring about other people and wanting them to be happy
- It depends, really distressed, bored, interpersonal/school stuff, stress, feeling trapped.
- during difficult time w/ her friends, there was a lot of stress on family because ggfather passed away
- Going through a lot, family troubles (grandpa getting sick/passing away), having no one to talk to during qurantine (she had only been talking to one person/her best friend from school). during difficult time w/ her friends, there was a lot of stress on family because ggfather passed away
- if I knew, I'd be better. probably because I just don't like myself at all. People being rude and saying stuff that's not very nice. Having problems with family and friends.

Psychosis

- feel something, dad, voices, emotions

Loss

- Family; grandma died; abusive father; "just really down"
- during difficult time w/ her friends, there was a lot of stress on family because father passed away
- Going through a lot, family troubles (grandpa getting sick/passing away), having no one to talk to during qurantine (she had only been talking to one person/her best friend from school). during difficult time w/ her friends, there was a lot of stress on family because grandfather passed away

Marginalized/ discrimination / rejection

- Bullying
- Bullying

Feeling like a burden

- Feel like a disappointment
- Feels like she deserves the pain, like she isn't good enough and deserves punishment

To impact others

- For attention and wanting someone to notice that I'm feeling down.
- "Thinking what will happen." "How my parents would feel and stuff
- other people are doing it (people around school and a friend)
- Sort of had friends who had engaged in self-harm, thing that crossed mind, a way to relieve stress
- a guy she knew talked to her about it - "that's somethings I could do"

Feel something/ address numbness

- wanting to feel something and feeling like deserve it
- To feeling something
- To feel something
- distraction, feel something
- to know I am still alive, feeling
- feel empty, try to feel ST [something], addicted to how it felt/having scars
- feel ST [something], dad, voices, emotions
- Bc physical pain takes away mental pain, don't really feel it
- Helps you not feel numb anymore, focus pain somewhere else
- Because she feels numb and wants to feel something
- As a way to release pain or emotion
- numb, felt like she needed to feel something.
- felt like she deserved it or needed to feel something
- self-worth, "I deserve this because I'm not good enough" - punishment. And a little trying to feel something
- Makes me feel something, forget or cope
- To feel something
- To feel something
- To feel something different
- She thinks it will make her feel better.
- Wanted to see if I could feel something.

- I don't know, probably just to feel something.
- I'm numb and I want to get out of it.
- I felt like I didn't feel anything, wanted to feel something
- Release feelings
- Feel relief
- I want to feel physical pain because I feel pain in my brain I (think?).
- To feel something, I have strong emotions- happen at different times
- it makes me feel less guilty, actually feel something. relieve emotional pain as physical pain
- Way she can feel pain without hurting other people.
- i can't keep up w/ what's going on, no other way for me to express what's going on inside. way of expressing the intensity of what's going on.
- it turns the mental pain into physical pain which is a lot easier to take in

Feel good
- Because it feels good

Becomes a habit
- Started out as a stress relief - now has "cravings" and "urges"
- A habit, miss it
- Don't know - just an urge
- just a habit now, wanted a way to not feel bad initially
- impulsive thing, in a bad mental spot

Other
- Different every time
- She wants to hurt herself but she doesn't want to die.
- Sort of had friends who had engaged in self-harm, thing that crossed mind, a way to relieve stress
- Bc physical pain takes away mental pain, don't really feel it
- Not really sure which is confusing, changed purposes show symptoms severe enough for treatment
- I don't really know, its cliche- so it cancels out the mental pain
- Idk exactly, one of therapists says ST because you need help but scared to tell. See cuts and get help. Probably relates to [her cutting]
- no idea, liked watching blood
- A habit, miss it
- Release endorphins
- Seemed like a way to help focus
- Way she can feel pain without hurting other people.
- For attention and wanting someone to notice that I'm feeling down.

Why do you think you engage in NSSI?

Don't Know
- I don't know
- Don't know, don't know what was running through, wasn't in fight with parents, don't remember
- I don't even know sometimes
- Unknown
- I don't know.

- IDK
- I don't know
- I don't even know.
- IDK
- doesn't know
- Unsure, going through a lot with friends
- I don't know
- I don’t know
- idk
- I don't know

Feeling sad / depressed / bad feelings – coping / relieve stress / pain

- Sad feelings, stressed
- to cope w/ feelings
- IDK to release pain
- Overwhelming
- Depression
- not to feel something
- To not feel sad
- to relieve stress
- Clears my thoughts/mind
- To let out emotions, emotional release
- Stress – release
- To release pain
- Really stressed
- A way to cope instead of talking
- Quiet down thoughts, feel things when not feeling well
- Stress, something (Dad) getting the best of
- Takes away from emotional pain
- the only thing she knew that would help (but clearly it didn't)
- As a way to cope
- Helps me cope, forget something
- Depressed
- Hard times, stress
- To get rid of bad feelings
- Because of mental state
- "Makes me feel better; gives you a different kind of pain to think about"
- Distracts in the mental pain
- impulsive, mad or distressed
- Stress reliever
- Escape bad feelings
- guilt, anger, frustration
- to make pain go away
- Get away from bad feelings, really intense feelings
- situational stress, depression
- I think because of the stress I have with homework (March)

- A way to get anger and stress out in a super intense moment.
- to relieve guilt, turn emotional pain into physical pain
- mostly school and mental state.
- Sadness
- to get away from bad feelings.
- get rid of bad feelings
- to distract herself
- to turn mental pain into physical pain
- Because she just feels really sad and hopeless, and it helps.

Becomes a Habit

- just a habit now, wanted a way to not feel bad initially
- A way to relieve stress, and then it becomes a habit.
- The urges were really strong, it's a habit;

Low self-esteem / Self-hatred / unhappiness with body / Punishment

- hate myself. deserve it. don't know explain it
- feel like deserve it
- self-hate, feel bad
- B/c dep.
- b/c I think I deserved it
- self-loathing
- When I do something wrong, it's sort of like a punishment, self-inflict punish, body image
- feel worthless
- feeling it was right thing, punishing self
- feeling of hating self, doing it b/c hate yourself but also hates self b/c of doing
- I was angry at myself and wanted to punish myself.
- Self-worth, not good enough for others or for anything. Punishment like I should be better
- Same as why she things about it because she deserves punishment
- punish self
- feel like I deserve it
- i deserve it.
- Frustrated with myself and wasn't doing good.; punishing myself / feeling like I deserved it
- because she feels like she did something wrong
- Punishment

Brain

- Release endorphins

Relationships

- Family problems; dad's abuse
- I had some falling out with friends (september)
- Problems with friends (went on a road trip without her) ended relationships.
- she feels like people doesn't love her.
- mom wasn't there, sister wasn't there either; no one to supervise; just "went wild" by being mean to myself

Psychosis

- Voices

An Escape / "only option"

- Escape
- Escape reality and find way out of things

Marginalized/ discrimination / rejection

- Bullying

To feel something / address numbness

- To be able to feel something
- Boredom(for nail digging)/ Boredom, stress, anxiety (for pulling)
- feel something
- to feel something, other than sadness or numbness
- To feel something
- feel something other than what feeling
- felt numb
- to feel something
- to feel something
- I want to feel something but I want to control the pain that I am feeling.
- to try to feel something
- Focus pain somewhere else, not feel numb anymore
- Because she feels numb
- because she felt like it was a breaking point - felt really sad and like she needed to do something to feel again
- feel something
- To feel something.
- Wanted to see if could feel something; So numb at the time.
- Cause I felt lonely or numb or really angry
- To feel something; to feel pain
- To feel something
- feel something
- To feel something
- wanted to feel something
- Felt guilty and bored and numb
- To feel something
- feel something
- to feel something.
- feeling so numb that I need to feel something,

To feel good

- To make myself feel better
- because in the moment it makes me feel better
- to stop thinking, focus on something else that gives me fake relief
- seeing scars after, addictive feeling, would feel better
- makes me feel better doing it

- soothing effect, short-term soothing and want to do more
- Thinks it will make her feel better.
- Because she likes to and she enjoys it.
- i think it's resolving the problem a little bit
- because the moment that you are doing it you don't think of anything else. Afterwards, you feel happy.

To impact others

- because mad at others and felt if hurt self it would hurt them
- Environmental factors- my friends, effects of it on friends
- For attention for telling people that "I'm serious" actually stop
- IDK - Probably thought it was only way for people to notice.
- Attention
- For attention

To prevent a suicide attempt

- to give myself a wakeup call to say - hey you're still alive and still got a fighting chance

Other

- Different every time
- Likes to see the blood
- Bc it would result in a feeling of clarity. Helped calm her thoughts
- Impulsivity
- Way to hurt herself without others noticing and drawing attention.
- form of expression, represent internal stress
- lots of different reasons

REFERENCES

18. Kaufman J, Birmaher B, Brent D, Rao UMA, Flynn C, Moreci P, et al. Schedule for affective disorders and schizophrenia for school-age children-present and lifetime version (K-SADS-PL): initial reliability and validity data. J Am Acad Child Adolesc Psychiatry. 1997;36(7):980–8. doi: 10.1097/00004583-199707000-00021

49. Flanagin A, Frey T, Christiansen SL, Committee AM of S. Updated guidance on the reporting of race and ethnicity in medical and science journals. Jama. 2021;326(7):621–7.

50. Association AP. APA Style: Racial and ethnic identity. September; 2019.

51. Yim IS, Granger DA, Quas JA. Children's and adults' salivary alpha-amylase responses to a laboratory stressor and to verbal recall of the stressor. Dev Psychobiol. 2010;52(6):598–602. doi: 10.1002/dev.20453

52. Yim IS, Quas JA, Rush EB, Granger DA, Skoluda N. Experimental manipulation of the Trier Social Stress Test-Modified (TSST-M) to vary arousal across development. Psychoneuroendocrinology. 2015;57:61–71. doi: 10.1016/j.psyneuen.2015.03.021

53. Kirschbaum C, Pirke KM, Hellhammer DH. The 'Trier Social Stress Test'–a tool for investigating psychobiological stress responses in a laboratory setting. Neuropsychobiology. 1993;28(1–2):76–81. doi: 10.1159/000119004

54. Klimes-Dougan B, Begnel E, Almy B, Thai M, Schreiner MW, Cullen KR. Hypothalamic-pituitary-adrenal axis dysregulation in depressed adolescents with non-suicidal self-injury. Psychoneuroendocrinology. 2019;102:216–24. doi: 10.1016/j.psyneuen.2018.11.004

55. Glasser MF, Coalson TS, Robinson EC, Hacker CD, Harwell J, Yacoub E, et al. A multi-modal parcellation of human cerebral cortex. Nature. 2016;536(7615):171–8. doi: 10.1038/nature18933

56. Hariri AR, Tessitore A, Mattay VS, Fera F, Weinberger DR. The amygdala response to emotional stimuli: a comparison of faces and scenes. Neuroimage. 2002;17(1):317–23. doi: 10.1006/nimg.2002.1179

57. Schneider W, Eschman A, Zuccolotto A. E-prime User’s guide., version pittsburgh: Psychology software tools inc. 2002;

58. Bates D, Mächler M, Bolker B, Walker S. Fitting linear mixed-effects models using lme4. ArXiv Prepr ArXiv14065823. 2014;

59. Kuznetsova A, Brockhoff PB, Christensen RH. lmerTest package: tests in linear mixed effects models. J Stat Softw. 2017;82:1–26. doi: 10.18637/jss.v082.i13
